# Galectin-8N-Selective 4-Halophenylphthalazinone-Galactals Double π-Stack in a Unique Pocket

**DOI:** 10.1021/acsmedchemlett.4c00212

**Published:** 2024-07-22

**Authors:** Sjors van Klaveren, Mujtaba Hassan, Maria Håkansson, Richard E. Johnsson, Jessica Larsson, Žiga Jakopin, Marko Anderluh, Hakon Leffler, Tihomir Tomašič, Ulf J. Nilsson

**Affiliations:** † Department of Chemistry, Faculty of Science, Lund University, Naturvetarvägen 14, 223 62, Lund, Sweden; ‡ SARomics Biostructures AB, Medicon Village, SE-223 81, Lund, Sweden; § Red Glead Discovery AB, Medicon Village, SE-223 81, Lund, Sweden; ∥ Pharmaceutical Chemistry, Faculty of Pharmacy, University of Ljubljana, Aškerčeva cesta 7, 1000 Ljubljana, Slovenia; ⊥ Department of Laboratory Medicine, Section MIG, Lund University, BMC-C1228b, Klinikgatan 28, 221 84, Lund, Sweden

**Keywords:** Galectin-8, Phthalazinone, Glycomimetic, X-ray, ADME

## Abstract

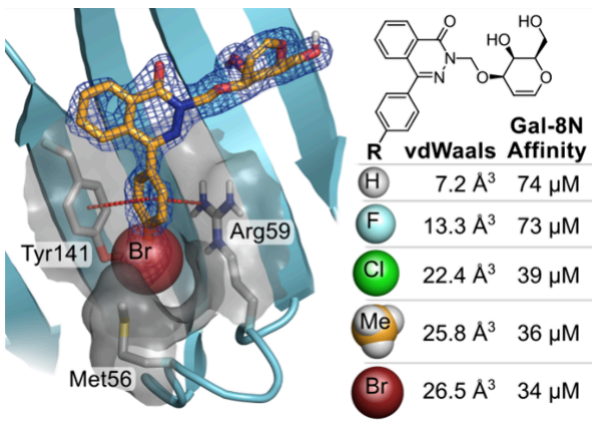

Galectin-8 contains two different carbohydrate recognition domains (CRDs). Selective inhibitors for at least one CRD are desirable for galectin-8 biology studies and potentially for pharmacological purposes. Structure-guided design led to the discovery of potent and selective glycomimetic–heterocycle hybrid ligands, with a 4-(*p*-bromophenyl)phthalazinone derivative displaying a 34 μM *K*_d_ for galectin-8N (N-terminal CRD), no binding to galectin-8C (C-terminal CRD), -1, -3, -4N, -7, -9C, or -9N, and >40-fold selectivity over galectin-4C. Selectivity was achieved with the halogenated 4-phenylphthalazinone moiety occupying a galectin-8N-specific sub-pocket. A 1.30 Å resolution X-ray structure revealed the phthalazinone moiety stacking with Arg45 and the 4-bromophenyl moiety stacking both Arg59 and Tyr141 of galectin-8N. Physicochemical and *in vitro* ADME studies revealed a desirable LogD, which also translated to good passive permeability. The chemical, microsome, and plasma stability support these compounds as promising tool compounds and candidates for hit-to-lead optimization.

Galectin-8 is a soluble galactose-binding lectin involved in cell–cell and cell–matrix signaling.^1,2^ Like other galectins, it is found both intra- and extracellularly and follows a non-traditional secretion pathway.^3,4^ Inside the cell, galectin-8N detects exposed glycans on ruptured vesicles to initiate antibacterial autophagy, and in a linked process, galectin-8C interacts in a protein–protein interaction with autophagy receptor NDP52.^5^ On the cell surface and in the extracellular matrix, it can interact with glycans of glycoproteins and engage in protein–protein interactions to initiate or enhance a wide range of signals.^6,7^ Several galectins interact with,^8^ and are exploited by,^9^ infecting bacterial and viral pathogens, including galectin-8.^10,11^ Healthy and pathogenic vascular angiogenesis and lymphangiogenesis are affected by galectin-8 and other galectins,^3^ through which they are associated with wound healing,^12^ organ graft rejection, and various diseases of the eye.^13^ Lymphangiogenesis also connects galectins to tumor progression^14^ and metastasis.^15^ Galectin-8 exacerbates this role by also stimulating cytokine secretion,^16^ which enhances tumor growth.^17^ Cytokine activity is related to several galectins,^18,19^ which underscores the intricate function of galectin-8 in the immune response.^20^ Galectin-8 has also been implicated in inflammatory disorders such as rheumatoid arthritis^21^ and, together with galectin-1, -4, and -9, in neurodegenerative diseases such as Alzheimer’s disease.^22^

The galectin-8 structure contains two different galectin carbohydrate recognition domains (CRDs), both β-sandwich domains of around 130 amino acids, connected via a linker of variable length (most commonly 32 amino acid residues).^1,2,23^ Although some galectin-8 functions are performed by each CRD independently,^23^ other biological roles require both CRDs and the linker,^24^ especially for interactions on the cell surface.^25^ Apart from binding galactoside ligands, galectins interact with target proteins via protein–protein interactions,^26,27^ and the best characterized example is the interaction of galectin-8C with cytosolic protein NDP52 mentioned above.^5^ Two crystal structures of galectin-8N in complex with a short peptide from NDP52 show a clearly defined binding site on the CRD (F-side) opposite from the carbohydrate binding site (S-side), independent of glycan recognition.^28,29^ This galectin-8–protein interaction appears to play a critical role in the autophagy of *Salmonella* bacteria.^30^ The possibility and biological effect of simultaneous protein and carbohydrate binding on galectin-8 remain to be studied and would require galectin-8-specific probes. Currently available fluorescent probes can distinguish the three binding sites on galectin-8: one for each galectin-8 carbohydrate binding-site^25^ and a peptide probe for the second site on galectin-8C.^29^ However, better selectivity over other galectins is needed.

For the galectin-8 functions that require the full-length protein, inhibition of either CRD is sufficient to interfere.^23^ Of the two galectin-8 CRDs, the N-terminal CRD has a higher affinity for its natural ligands.^25^ For this reason, galectin-8N has been the primary target for novel glycomimetic ligands.^31^ These ligands have had increasing galectin-8N affinity and selectivity over other galectins.^32^ One tricyclic glycomimetic scaffold had already relinquished a part of the galactose sugar stereochemistry by fusing a benzene ring to create a 180 μM ligand for galectin-8N with a 2-fold selectivity over galectin-1.^33^ Other inhibitors include promising O3-carboxyethyl galactoside or O3-galactomalonyl phenyl esters, with detailed thermodynamics (i.e., isothermal calorimetry)^34^ characterization and promising galectin-8N inhibition in cell lines,^35^ but no reported selectivity over other galectins.

Among the most recently published ligands from our group are the d-galactal-phthalazinone conjugates **1** and **2**,^36^ which showed a high selectivity for galectin-8N. Of these two compounds, 4-phenylphthalazinone **2** had a slightly higher affinity and was more selective due to the position of the phenyl in a galectin-8N-specific sub-pocket, as inferred from molecular dynamics (MD) simulations (Figure 1). One side of this sub-pocket comprises residues 54–59 of the flexible S3–S4 loop, which creates a potential space for an additional functionalization of the phenyl group. In this work, we present optimized ligands that target this potential space to achieve almost complete selectivity for galectin-8N over other tested human galectins.

**Figure 1:**
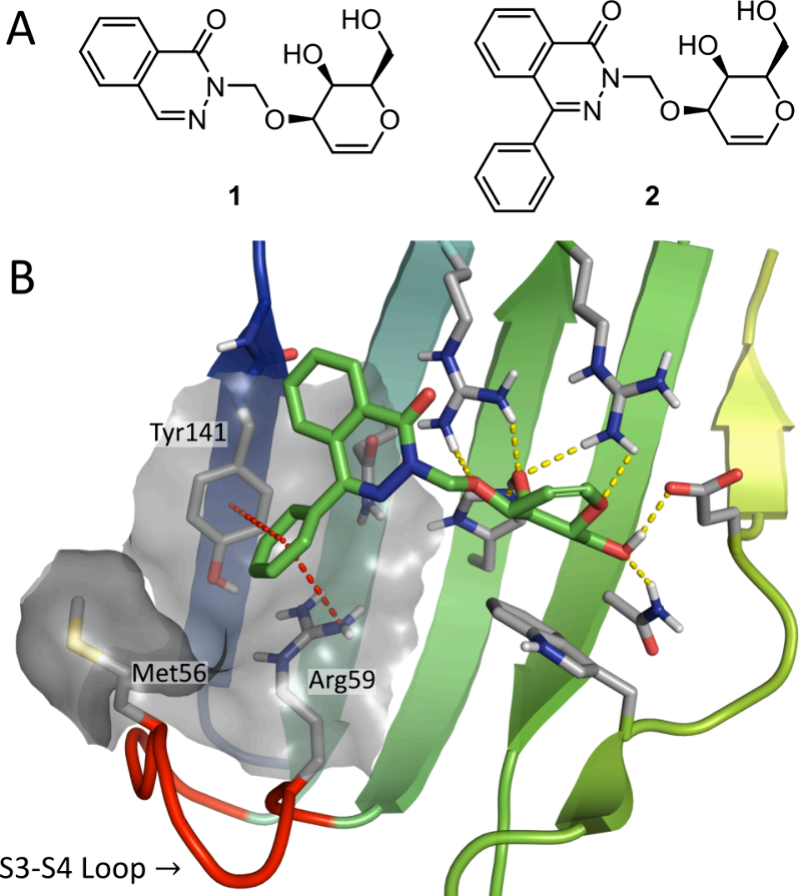
A) Known phthalazinone-galactal ligands of galectin-8N. B) Molecular dynamics snapshot^36^ of compound **2** shown with the ligand in green sticks, hydrogen bonds in yellow dotted lines, and π–π stacking with Tyr141 and cation−π interactions with Arg59 to the phenyl group shown in red dotted lines. The flexible S3–S4 loop is colored red. The protein surface around residues Met56, Arg59, and Tyr141 is included to visualize the targeted sub-pocket. (Figure was generated with PyMOL v1.7, Schrodinger LLC.)

## Synthesis

**Scheme 1:**
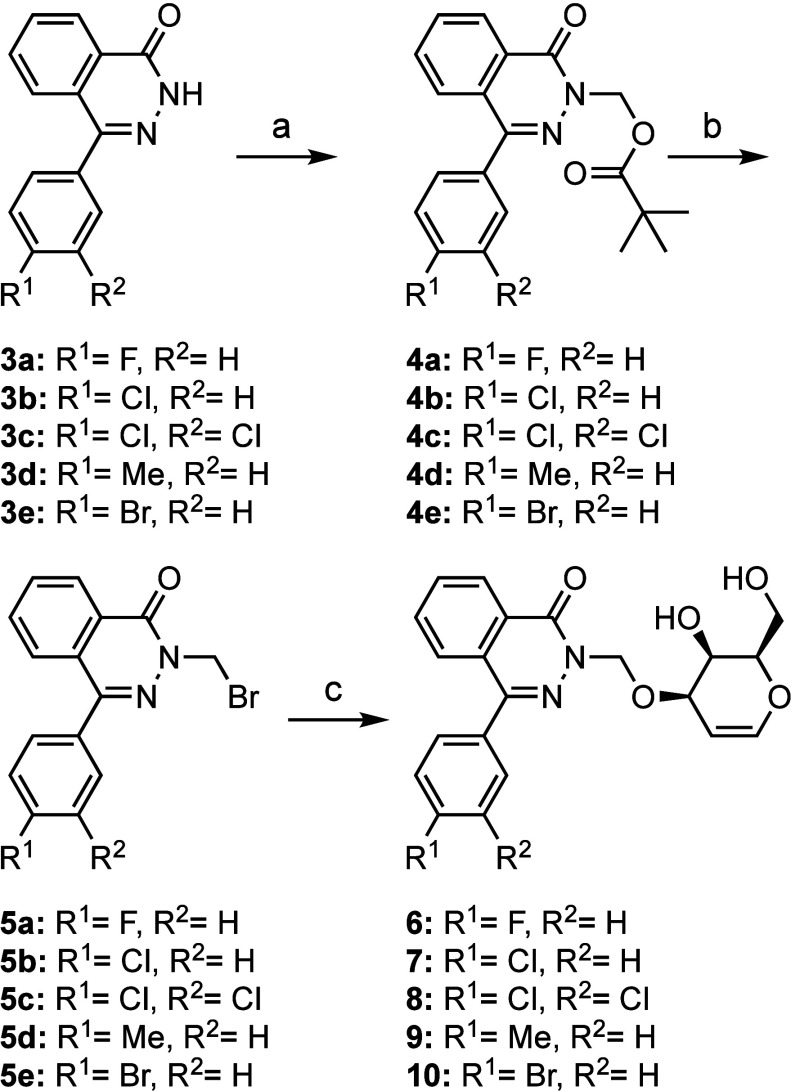
Synthesis of Functionalized Galactal-Phthalazinones Reagents and conditions: (a) for compounds **3a**, **3d**, and **3e**, chloromethyl pivalate, Cs_2_CO_3_, DMF, 70 °C, 1.5 h (65–90% yield); for compounds **3b** and **3c**, chloromethyl pivalate, NaH, THF, reflux, 1.5 h (95–96% yield); (b) HBr (33% wt in AcOH), AcOH, 75 °C, 1 h (79–98% yield); (c) d-galactal, K_2_CO_3_, *n*-Bu_2_SnO, TBAI, MeCN/Tol, 30 min, 120 °C (18–40% yield after prep. chromatography).

The synthesis follows an approach analogous to that used for d-galactal-phthalazinone structures **1** and **2** published previously (Scheme 1).^36^ Phthalazinones **3b** and **3c** were not commercially available but could be efficiently prepared by hydrazine cyclo-condensation of the relevant 2-benzoylbenzoic acids (General procedure A).^37^ Phenylphthalazinone structures **3a**–**e** were *N*-alkylated in basic conditions (Scheme 1). Two methods were used: cesium carbonate in *N*,*N*-dimethylformamide (DMF) and sodium hydride in tetrahydrofuran (THF); the latter method was preferred because of the higher yields and easier work-up. The resulting *N*-methyl pivalate intermediates **4a**–**e** were brominated with hydrogen bromide to give *N*-methyl bromides **5a**–**e**. For the nucleophilic attack of d-galactal on methyl bromide **5**, the O3 of d-galactal was activated by *in situ* formation of a 3,4-dibutylstannylene-acetal intermediate. This activation facilitates a selective alkylation of galactal-O3 to give compounds **6**–**10**. The final molecules present different halogens and a methyl on the phenyl 4-position, as well as 3,4-dichloro compound **8**.

## Galectin Binding Data

The affinities of compounds **1** and **2** and the novel compounds **6**–**10** for human galectin-1, -3, -4C, -4N, -7, -8C, -8N, -9C, and -9N were determined in a competitive fluorescence anisotropy assay (Table 1). Some of the ligands showed moderate (∼1 mM) affinity for other galectins, but the overall selectivity of the galactal–phthalazinones for the target galectin-8N is upheld. None of the functionalized phenylphthalazinones (**6**–**10**) shows any affinity for galectin-4N, -7, and -9N, and only low affinity (∼2 mM) is observed for galectin-1 and -9C. The *p*-methylphenyl **9** shows a relatively high 36 μM affinity for galectin-8N, but its higher affinity for the other tested galectins (-8C, -3, and -4C) makes it the least selective ligand. All ligands show a weak affinity for galectin-4C.

**Table 1:** *K*_d_ Values (μM ± SEM)^a^ Obtained by Fluorescence Anisotropy Assay of Phthalazinones **1**, **2**, **6**–**10**

	Galectin								
	8N	8C	1	3	4C	4N	7	9C	9N
**1** -H^36^	86 ± 5.2	2600 ± 220^c^	2100 ± 370^c^	2900 ± 410^c^	∼1800^d^	1300 ± 20^c^	N.B.^b^	2400 ± 240^c^	N.B.^b^
**2** Ph-H^36^	74 ± 1.2	2700 ± 58^c^	3000 ± 530^c^	N.B.^b^	∼2200^d^	960 ± 110^c^	N.B.^b^	2600 ± 310^c^	N.B.^b^
**6** Ph-F	73 ±1.7	1700 ± 150^c^	N.B.^b^	∼2200^d^	1500 ± 170^c^	N.B.^b^	N.B.^b^	2200 ± 250^c^	N.B.^b^
**7** Ph-Cl	39 ± 1.7	3100 ± 310^c^	N.B.^b^	N.B.^b^	1600 ± 200^c^	N.B.^b^	N.B.^b^	N.B.^b^	N.B.^b^
**8** Ph-Cl_2_	180 ± 39	N.B.^b^	N.B.^b^	N.B.^b^				N.B.^b^	N.B.^b^
**9** Ph-Me	36 ± 1.7	960 ± 64	1900 ± 300^c^	1100 ± 90^c^	1100 ± 320	N.B.^b^	N.B.^b^	N.B.^b^	N.B.^b^
**10** Ph-Br	34 ± 3.2	N.B.^b^	N.B.^b^	N.B.^b^	1400 ± 130^c^	N.B.^b^	N.B.^b^	N.B.^b^	N.B.^b^

a If not stated otherwise, results represent the mean ± SEM of *n* = 4 to 8.

b Non-binding up to the highest tested concentration of 1200–1500 μM.

c Affinity based on two data points at the highest concentration (≥ 1200 μM).

d Standard error not available; affinity approximated from one data point at the highest concentration (≥1200 μM).

For galectin-8N, the *p*-chlorophenyl **7** and the *p*-bromophenyl **10** have within error the same *K*_d_ as the *p*-methylphenyl **9**. The affinities of **7** and **10** are 41-fold better than their *K*_d_ values for galectin-4C, with no affinity measured for any of the other tested galectins (at concentrations up to 1500 μM). Thus, 4-(*p*-chlorophenyl)phthalazinone **7** and 4-(*p*-bromophenyl)phthalazinone **10** are the most selective galectin-8N ligands reported to date. The commonly used galectin inhibitors, lactose and thiodigalactoside, have *K*_d_ values of 91 and 61 μM for galectin-8N and virtually no selectivity among the galectins.^25,38^ Compounds **7** and **10**, despite their moderate 3–4-fold affinity enhancements over lactose, represent significantly better chemical biology tool compounds for addressing galectin-8N biology.

**Figure 2:**
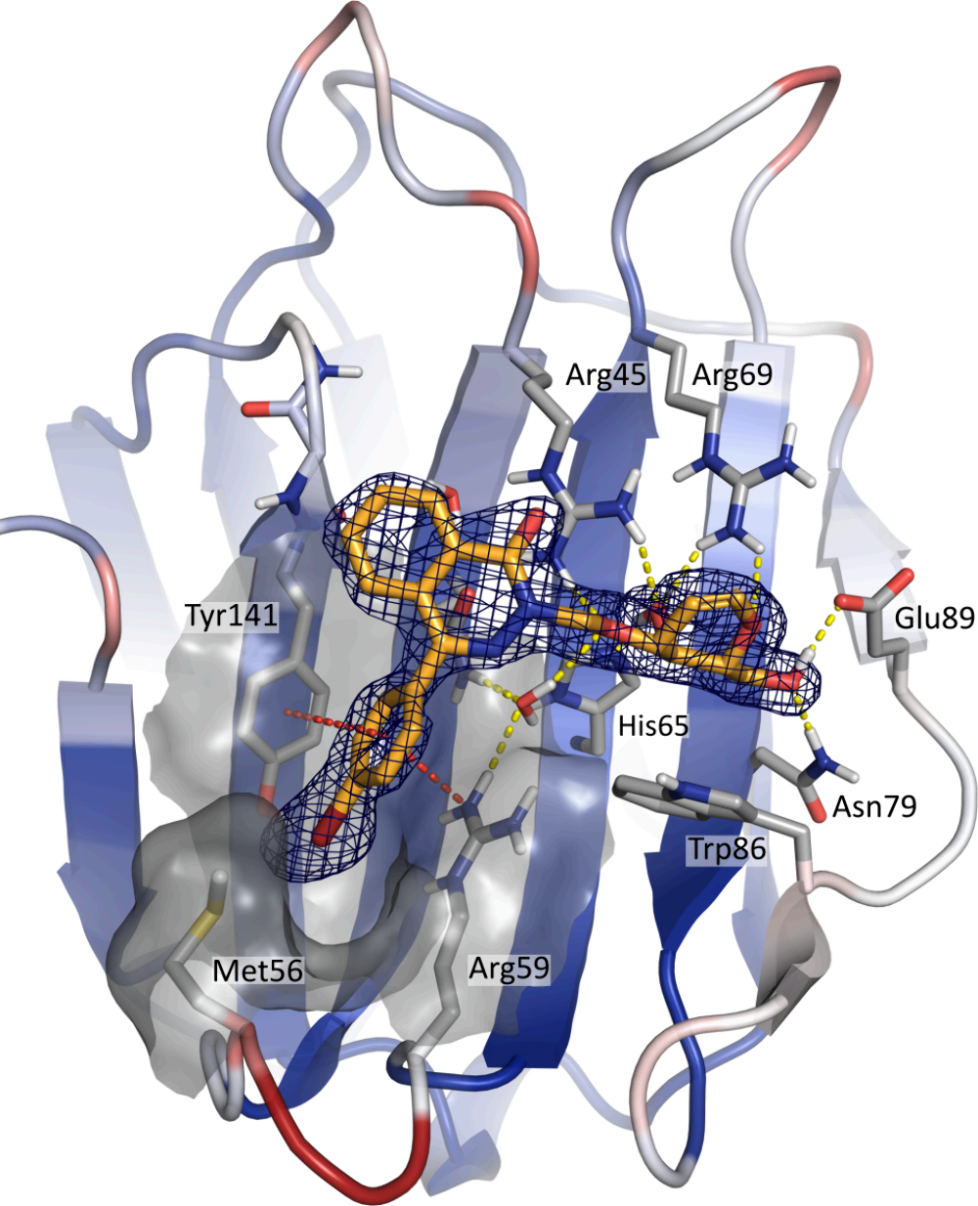
Crystal structure of galectin-8N-galactal-phthalazinone **10** with the electron density map of the ligand as a dark blue mesh, 2|*F*_o_| – |*F*_c_|, α*c* contoured at 1σ for the best occupied site (chain B). Ligand presented in orange sticks; protein colored in red-white-blue according to B-factor, showing the flexibility of the S3–S4 loop. Hydrogen bonding network is presented with yellow dotted lines; cation−π and π–π stacking are presented with red dotted lines (PDB ID: 9FXZ). (Figure was generated with PyMOL v1.7, Schrodinger LLC.)

On the flexible S3–S4 loop of galectin-8N, the amino acid Met56 can also occur as Val56—a natural variant with no disease association—of which X-ray crystal structures have also been deposited in the RSCB-Protein Data Bank (14 structures with Val56 and 13 structures with Met56, thus far). As the residue in this position is part of the sub-pocket surrounding the halogenated phenyl, its significance in the binding of these ligands was investigated. The binding affinities obtained in comparative fluorescence polarization assays revealed that the binding affinities of compounds **1**, **2**, **9**, and **10** for galectin-8N Met56 variant and galectin-8N Val56 variant (Table S1) only differed marginally. The similar affinities indicate that galectin-8N ligands exploiting the mentioned sub-pocket, whether research probes or drug candidates, can be used regardless of variant. In Table 1, galectin-8N Met56 variant affinity data are given as it aligns with the presented X-ray crystal structure (Figure 2).

## Structural Analysis

To assess the binding mode of halogenated phenylphthalazinones, we solved the X-ray crystal structure of galectin-8N (Met56) in complex with **10** at a resolution of 1.30 Å (PDB ID: 9FXZ) with two molecules in the asymmetric unit. The complex was obtained by soaking 2 mM inhibitor **10** into crystals of the galectin-8N-lactose complex.^32^ The inhibitor **10** has been modeled with similar conformation and partial occupancy (0.5 in chain A and 0.7 in chain B) in both sites to best fit the electron density maps (Table S2).

The crystal complex reveals that the binding mode of *p*-bromophenyl derivative **10** corresponds to that predicted for the 4-phenyl analogue **2** by MD simulations (see Supporting Information, Figure S1).^36^ The d-galactal moiety is localized in the galactose binding region (Figure 2), and the 4-hydroxyl group establishes a concerted hydrogen-bonding network with Arg45, His65, and Arg69, while the 6-hydroxyl group binds to Asn79 and Glu89. The hydrophobic α-side of the d-galactal is stacking to the surface of Trp86. This position aligns with previous galectin-8N complexes with d-galactal conjugates (PDB ID: 7P11).^39^

The electron density detected in the sub-pocket reveals the position of the *p*-bromophenyl substituent. In this pocket, it is in range to engage in a cation−π stacking with Arg59 and a π–π T-stacking with Tyr141. The bromide atom is placed close-fitting near Met56, Tyr141, and the backbone of the flexible S3–S4 loop. The heterocyclic ring of the phthalazinone is positioned in a suitable range and direction to engage in cation−π stacking with Arg45.

A slightly weaker electron density around the phthalazinone carbons suggests some motility of this part of the molecule. This motility can be explained using earlier observations made in MD simulations of phthalazinone inhibitor **2** in the CRD of galectin-8N, which recognized an alternative possible binding mode during a 1 μs MD simulation.^36^ However, this alternative binding mode was not recognized in the electron density map (Figure 2) suggesting that, in crystallography settings, it is not a favorable conformation.

## Structure–Affinity Relationships

Surprisingly, the electronic nature of the halogen substituents does not directly dictate the structure–affinity relationship, as exemplified by the similar affinity of *p*-methylphenyl **9** and *p*-bromophenyl **10**. The relative size of these two substituents, however, is quite similar. The van der Waals volume of the corresponding space group on the phenylphthalazinone core is given in Table 2, alongside the respective affinity for galectin-8N. The increasing size of substituents correlates to the increase in affinity (*R*^2^ = 0.90) and might be the result of suitably occupying the sub-pocket between Met56, Tyr141, and Arg59 (Figure 1).

**Table 2:** Comparison of the Phenyl *p*-Substituent van der Waals Volumes and *K*_d_ Values (μM) Obtained by the Fluorescence Anisotropy Assay

	Atom	vdW volume (Å^3^)^40^	Gal-8N affinity (μM)
**2**	H	7.24	74
**6**	F	13.31	73
**7**	Cl	22.45	39
**9**	Me	25.83	36
**10**	Br	26.52	34

## Physicochemical Properties

Several physicochemical and ADME properties of selected galactal-phthalazinones were evaluated *in vitro* (Table S3). Solubility screening was assessed at a 100 μM concentration in phosphate-buffered saline (10 mM, pH 7.4). After 24 h of continuous perturbation at room temperature, LC-UV analysis revealed that most compounds are entirely solubilized, except for the *p*-chlorophenyl derivative **7** and the *p*-bromophenyl derivative **10** (Table S4). The *p*-fluorophenyl derivative (**9**) also shows slightly lower solubility (94% vs >95% for **2**). Hence, replacing a *p*-H (**2**) with *p*-halogens (**6**, **7**, and **10**) of increasing size leads, not unexpectedly, to decreasing solubility, while chloro isosteric methyl **9** retains good solubility. The experimental LogD was obtained by LC-UV analysis of the compound distribution between 1-octanol and phosphate buffer (10 mM, pH 7.4, Table S5). A partial correlation between solubility and LogD exists, as the least soluble chloro (**7**) and bromo (**10)** derivatives also have higher LogD. However, the methyl **9** breaks the correlation with a high LogD despite having high solubility. Chemical stability was assessed, after 5 days of continuous mixing in acidic (pH 4) or basic (pH 9.5) ammonium acetate buffer, by the presence or absence of degradation products in LC-UV analysis. All compounds were stable under these conditions (Table S6).

## *In Vitro* ADME Properties

The blood plasma stability of most compounds was over 30 h, which is within desirable parameters (Table S7). The exception was the *p*-methylphenyl derivative **9**, which suggests that the methylphenyl group of **9** is degraded by plasma proteins. The microsomal stability of compounds was assessed with both mouse and human liver microsomes (Tables S8 and S9). For all phenylphthalazinones, the measured mouse microsome stability is in the low range, although the halogenated compounds fared slightly better. The human microsome stability was high, indicating that the molecule is not directly degraded by human liver enzymes. While human microsome stability is critical in drug development, low mouse microsomal stability must be considered in preclinical mouse models. A parallel artificial membrane permeability assay (PAMPA) was performed with compounds **1**, **2**, **6**, and **10** to determine the apparent passive permeability. The apparent permeability is high (>5) for all compounds (Table S10), which indicates that passive permeability is to be expected *in vivo*.

In conclusion, optimized glycomimetic ligands have been synthesized with increased affinity for the target galectin-8N and an unmatched selectivity profile over human galectin-1, -3, -4C, -4N, -7, -8C, -9C, and -9N. The highest affinity ligand, the 4-(*p*-bromophenyl)phthalazinone-galactal **10**, in complex with galectin-8N was studied with X-ray crystallography to elucidate the binding pose and interactions and rationalize the structure–activity relationship. This structure reveals the phenyl group engages in cation−π stacking and π–π T-stacking in a galectin-8N specific sub-pocket, and the *para*-bromide is favorably fitted among residues of the flexible S3–S4 loop, leading to the selectivity for galectin-8N as other galectins lack a corresponding pocket.

Based on the in vitro physicochemical and ADME studies, the galactal-phenylphthalazinones are interesting compounds for hit-to-lead development. The chemical stability of the compounds is good, and they have desirable lipophilicity (LogD) and passive permeability properties. Suitable pharmacokinetics in humans are expected based on solubility, permeability, and *in vitro* stability in blood plasma and human liver microsome studies. The most selective ligand, the *p*-bromophenylphthalazinone **10**, showed a lower solubility, which, however, may be improved by including a hydrophilic/ionizable group, such as a carboxylic acid that previously improved the affinities of other ligands for galectin-8N.^32^

The resulting structural and ADME data provide information for the future hit-to-lead development of galectin ligands with pharmaceutical application. The high selectivity of the halogenated galactal-phenylphthalazinones for galectin-8N makes them a valuable molecular tool for studying galectin-8 biology and unraveling the variety of galectin-8-associated pathologies.

## Supporting Information Available

^1^H NMR and ^13^C NMR spectra, HLPC traces and analyses, and X-ray crystallography data collection and refinement statistics; comparative affinity analysis of compounds **1**, **2**, **9**, and **10** for galectin-8N (Met56)/Galectin-8N (Val56); methods and data for the screen solubility, chemical stability, LogD, plasma stability, mouse liver microsomal stability, human liver microsomal stability, and PAMPA (PDF)

## Abbreviations

CRD: carbohydrate recognition domain
N: N-terminal
C: C-terminal
MD: molecular dynamics
TLC: thin-layer chromatography

## References

[ref1] Bidon-Wagner N.; Le Pennec J.-P. Human Galectin-8 Isoforms and Cancer. Glycoconj. J. 2002, 19 (7-9), 557–563. 10.1023/B:GLYC.0000014086.38343.98. 14758080

[ref2] Johannes L.; Jacob R.; Leffler H. Galectins at a Glance. J. Cell Sci. 2018, 131 (9), jcs208884 10.1242/jcs.208884. 29717004

[ref3] Troncoso M. F.; Ferragut F.; Bacigalupo M. L.; Delgado V. M. C.; Nugnes L. G.; Gentilini L.; Laderach D.; Wolfenstein-Todel C.; Compagno D.; Rabinovich G. A.; Elola M. T. Galectin-8: A Matricellular Lectin with Key Roles in Angiogenesis. Glycobiology 2014, 24 (10), 907–914. 10.1093/glycob/cwu054. 24939370

[ref4] Bänfer S.; Jacob R. Galectins in Intra-and Extracellular Vesicles. Biomolecules 2020, 10 (9), 1232 10.3390/biom10091232. 32847140 PMC7563435

[ref5] Thurston T. L. M.; Wandel M. P.; Von Muhlinen N.; Foeglein Á.; Randow F. Galectin 8 Targets Damaged Vesicles for Autophagy to Defend Cells against Bacterial Invasion. Nature 2012, 482 (7385), 414–418. 10.1038/nature10744. 22246324 PMC3343631

[ref6] Compagno D.; Jaworski F. M.; Gentilini L.; Contrufo G.; Perez I.; Elola M. T.; Pregi N.; Rabinovich G. A.; Laderach D. J. Galectins: Major Signaling Modulators Inside and Outside the Cell. Curr. Mol. Med. 2014, 14 (5), 630–651. 10.2174/1566524014666140603101953. 24894174

[ref7] Elola M. T.; Blidner A. G.; Ferragut F.; Bracalente C.; Rabinovich G. A. Assembly, Organization and Regulation of Cell-Surface Receptors by Lectin-Glycan Complexes. Biochem. J. 2015, 469 (1), 1–16. 10.1042/BJ20150461. 26173257

[ref8] Lin C. Y.; Yang Z. S.; Wang W. H.; Urbina A. N.; Lin Y. T.; Huang J. C.; Liu F. T.; Wang S. F. The Antiviral Role of Galectins toward Influenza a Virus Infection—an Alternative Strategy for Influenza Therapy. Pharmaceuticals 2021, 14 (5), 490 10.3390/ph14050490. 34065500 PMC8160607

[ref9] Ayona D.; Fournier P.-E. E.; Henrissat B.; Desnues B. Utilization of Galectins by Pathogens for Infection. Front. Immunol. 2020, 11, 1877 10.3389/fimmu.2020.01877. 32973776 PMC7466766

[ref10] Wang W. H.; Lin C. Y.; Chang M. R.; Urbina A. N.; Assavalapsakul W.; Thitithanyanont A.; Chen Y. H.; Liu F. T.; Wang S. F. The Role of Galectins in Virus Infection - A Systemic Literature Review. J. Microbiol. Immunol. Infect. 2020, 53 (6), 925–935. 10.1016/j.jmii.2019.09.005. 31630962

[ref11] Liu F. T.; Stowell S. R. The Role of Galectins in Immunity and Infection. Nat. Rev. Immunol. 2023, 23 (8), 479–494. 10.1038/s41577-022-00829-7. 36646848 PMC9842223

[ref12] Yu D.; Bu M.; Yu P.; Li Y.; Chong Y. Regulation of Wound Healing and Fibrosis by Galectins. J. Mol. Med. 2022, 100 (6), 861–874. 10.1007/s00109-022-02207-1. 35589840

[ref13] Chen W. S.; Cao Z.; Sugaya S.; Lopez M. J.; Sendra V. G.; Laver N.; Leffler H.; Nilsson U. J.; Fu J.; Song J.; Xia L.; Hamrah P.; Panjwani N. Pathological Lymphangiogenesis Is Modulated by Galectin-8-Dependent Crosstalk between Podoplanin and Integrin-Associated VEGFR-3. Nat. Commun. 2016, 7, 1–17. 10.1038/ncomms11302. PMC4832077 27066737

[ref14] Thijssen V. L.; Rabinovich G. A.; Griffioen A. W. Vascular Galectins: Regulators of Tumor Progression and Targets for Cancer Therapy. Cytokine Growth Factor Rev. 2013, 24 (6), 547–558. 10.1016/j.cytogfr.2013.07.003. 23942184

[ref15] Girotti M. R.; Salatino M.; Dalotto-Moreno T.; Rabinovich G. A. Sweetening the Hallmarks of Cancer: Galectins as Multifunctional Mediators of Tumor Progression. J. Exp. Med. 2020, 217 (2), e20182041 10.1084/jem.20182041. 31873723 PMC7041721

[ref16] Gordon-Alonso M.; Bruger A. M.; van der Bruggen P. Extracellular Galectins as Controllers of Cytokines in Hematological Cancer. Blood 2018, 132 (5), 484–491. 10.1182/blood-2018-04-846014. 29875102 PMC6073326

[ref17] Shatz-Azoulay H.; Vinik Y.; Isaac R.; Kohler U.; Lev S.; Zick Y. The Animal Lectin Galectin-8 Promotes Cytokine Expression and Metastatic Tumor Growth in Mice. Sci. Rep. 2020, 10 (1), 7375 10.1038/s41598-020-64371-z. 32355198 PMC7193594

[ref18] Porębska N.; Poźniak M.; Matynia A.; Żukowska D.; Zakrzewska M.; Otlewski J.; Opaliński Ł. Galectins as Modulators of Receptor Tyrosine Kinases Signaling in Health and Disease. Cytokine Growth Factor Rev. 2021, 60, 89–106. 10.1016/j.cytogfr.2021.03.004. 33863623

[ref19] Sanjurjo L.; Broekhuizen E. C.; Koenen R. R.; Thijssen V. L. J. L. Galectokines: The Promiscuous Relationship between Galectins and Cytokines. Biomolecules 2022, 12 (9), 1286 10.3390/biom12091286. 36139125 PMC9496209

[ref20] Tribulatti M. V.; Carabelli J.; Prato C. A.; Campetella O. Galectin-8 in the Onset of the Immune Response and Inflammation. Glycobiology 2020, 30 (3), 134–142. 10.1093/glycob/cwz077. 31529033

[ref21] Massardo L.; Metz C.; Pardo E.; Mezzano V.; Babul M.; Jarpa E.; Guzmán A. M.; André S.; Kaltner H.; Gabius H. J.; Jacobelli S.; González A.; Soza A. Autoantibodies against Galectin-8: Their Specificity, Association with Lymphopenia in Systemic Lupus Erythematosus and Detection in Rheumatoid Arthritis and Acute Inflammation. Lupus 2009, 18 (6), 539–546. 10.1177/0961203308099973. 19395456

[ref22] Ramírez Hernández E.; Alanis Olvera B.; Carmona González D.; Guerrero Marín O.; Pantoja Mercado D.; Valencia Gil L.; Hernández-Zimbrón L. F.; Sánchez Salgado J. L.; Limón I. D.; Zenteno E. Neuroinflammation and Galectins: A Key Relationship in Neurodegenerative Diseases. Glycoconj. J. 2022, 39 (5), 685–699. 10.1007/s10719-022-10064-w. 35653015

[ref23] Cagnoni A. J.; Troncoso M. F.; Rabinovich G. A.; Mariño K. V.; Elola M. T. Full-Length Galectin-8 and Separate Carbohydrate Recognition Domains: The Whole Is Greater than the Sum of Its Parts?. Biochem. Soc. Trans. 2020, 48 (3), 1255–1268. 10.1042/BST20200311. 32597487

[ref24] Si Y.; Cai J.; Zhu J.; Wang Y.; Zhang F.; Meng L.; Huang J.; Shi A. Linker Remodels Human Galectin-8 Structure and Regulates Its Hemagglutination and pro-Apoptotic Activity. Int. J. Biol. Macromol. 2023, 245, 125456 10.1016/j.ijbiomac.2023.125456. 37331541

[ref25] Carlsson S.; Öberg C. T.; Carlsson M. C.; Sundin A.; Nilsson U. J.; Smith D.; Cummings R. D.; Almkvist J.; Karlsson A.; Leffler H. Affinity of Galectin-8 and Its Carbohydrate Recognition Domains for Ligands in Solution and at the Cell Surface. Glycobiology 2007, 17 (6), 663–676. 10.1093/glycob/cwm026. 17339281

[ref26] Meinohl C.; Barnard S. J.; Fritz-Wolf K.; Unger M.; Porr A.; Heipel M.; Wirth S.; Madlung J.; Nordheim A.; Menke A.; Becker K.; Giehl K. Galectin-8 Binds to the Farnesylated c-Terminus of k-Ras4b and Modifies Ras/Erk Signaling and Migration in Pancreatic and Lung Carcinoma Cells. Cancers (Basel). 2020, 12 (1), 30 10.3390/cancers12010030. PMC7017085 31861875

[ref27] Tribulatti M. V.; Cattaneo V.; Hellman U.; Mucci J.; Campetella O. Galectin-8 Provides Costimulatory and Proliferative Signals to T Lymphocytes. J. Leukoc. Biol. 2009, 86 (2), 371–380. 10.1189/jlb.0908529. 19401394

[ref28] Li S.; Wandel M. P.; Li F.; Liu Z.; He C.; Wu J.; Shi Y.; Randow F. Sterical Hindrance Promotes Selectivity of the Autophagy Cargo Receptor NDP52 for the Danger Receptor Galectin-8 in Antibacterial Autophagy. Sci. Signal. 2013, 6 (261), ra9 10.1126/scisignal.2003730. 23386746 PMC3713453

[ref29] Kim B. W.; Beom Hong S.; Hoe Kim J.; Hoon Kwon D.; Song H. K. Structural Basis for Recognition of Autophagic Receptor NDP52 by the Sugar Receptor Galectin-8. Nat. Commun. 2013, 4, 1613 10.1038/ncomms2606. 23511477

[ref30] Kwon D. H.; Song H. K. A Structural View of Xenophagy, a Battle between Host and Microbes. Mol. Cells 2018, 41 (1), 27–34. 10.14348/MOLCELLS.2018.2274. 29370690 PMC5792709

[ref31] Pal K. B.; Mahanti M.; Huang X.; Persson S.; Sundin A. P.; Zetterberg F. R.; Oredsson S.; Leffler H.; Nilsson U. J. Quinoline-Galactose Hybrids Bind Selectively with High Affinity to a Galectin-8 N-Terminal Domain. Org. Biomol. Chem. 2018, 16 (34), 6295–6305. 10.1039/C8OB01354C. 30117507

[ref32] Hassan M.; Van Klaveren S.; Håkansson M.; Diehl C.; Kovačič R.; Baussière F.; Sundin A. P.; Dernovšek J.; Walse B.; Zetterberg F.; Leffler H.; Anderluh M.; Tomašič T.; Jakopin Ž.; Nilsson U. J. Benzimidazole-Galactosides Bind Selectively to the Galectin-8 N-Terminal Domain: Structure-Based Design and Optimisation. Eur. J. Med. Chem. 2021, 223, 113664 10.1016/j.ejmech.2021.113664. 34225180

[ref33] Wu C.; Yong C.; Zhong Q.; Wang Z.; Nilsson U. J.; Zhang Y. Synthesis of Tricyclic Carbohydrate-Benzene Hybrids as Selective Inhibitors of Galectin-1 and Galectin-8 N-Terminal Domains. RSC Adv. 2020, 10 (33), 19636–19642. 10.1039/D0RA03144E. 35515421 PMC9054096

[ref34] Bohari M. H.; Yu X.; Kishor C.; Patel B.; Go R. M.; Eslampanah Seyedi H. A.; Vinik Y.; Grice I. D.; Zick Y.; Blanchard H. Structure-Based Design of a Monosaccharide Ligand Targeting Galectin-8. ChemMedChem. 2018, 13 (16), 1664–1672. 10.1002/cmdc.201800224. 29926535

[ref35] Patel B.; Kishor C.; Houston T. A.; Shatz-Azoulay H.; Zick Y.; Vinik Y.; Blanchard H. Rational Design and Synthesis of Methyl-β- d -Galactomalonyl Phenyl Esters as Potent Galectin-8 N Antagonists. J. Med. Chem. 2020, 63 (20), 11573–11584. 10.1021/acs.jmedchem.0c00602. 32809817

[ref36] Van Klaveren S.; Sundin A. P.; Jakopin Ž.; Anderluh M.; Leffler H.; Nilsson U. J.; Tomašič T. Selective Galectin-8N Ligands: The Design and Synthesis of Phthalazinone-D-Galactals. ChemMedChem. 2022, 17 (6), e202100575 10.1002/cmdc.202100575. 34913595

[ref37] Terán C.; Besada P.; Vila N.; Costas-Lago M. C. Recent Advances in the Synthesis of Phthalazin-1(2H)-One Core as a Relevant Pharmacophore in Medicinal Chemistry. Eur. J. Med. Chem. 2019, 161, 468–478. 10.1016/j.ejmech.2018.10.047. 30388463

[ref38] Cumpstey I.; Salomonsson E.; Sundin A.; Leffler H.; Nilsson U. J. Double Affinity Amplification of Galectin-Ligand Interactions through Arginine-Arene Interactions: Synthetic, Thermodynamic, and Computational Studies with Aromatic Diamido Thiodigalactosides. Chem. - A Eur. J. 2008, 14 (14), 4233–4245. 10.1002/chem.200701932. 18366047

[ref39] Hassan M.; Baussière F.; Guzelj S.; Sundin A. P.; Håkansson M.; Kovačič R.; Leffler H.; Tomašič T.; Anderluh M.; Jakopin Ž.; Nilsson U. J. Structure-Guided Design of d -Galactal Derivatives with High Affinity and Selectivity for the Galectin-8 N-Terminal Domain. ACS Med. Chem. Lett. 2021, 12 (11), 1745–1752. 10.1021/acsmedchemlett.1c00371. 34795863 PMC8592027

[ref40] Zhao Y. H.; Abraham M. H.; Zissimos A. M. Fast Calculation of van Der Waals Volume as a Sum of Atomic and Bond Contributions and Its Application to Drug Compounds. J. Org. Chem. 2003, 68 (19), 7368–7373. 10.1021/jo034808o. 12968888

