# Supplementary material for: Galectin-8N-Selective 4-Halophenylphthalazinone-Galactals Double π-Stack in a Unique Pocket
Source: ACS Med Chem Lett. 2024 Jul 22;15(8):1319–24. doi: 10.1021/acsmedchemlett.4c00212 (PMC11318003; doi:10.1021/acsmedchemlett.4c00212)
Supplement: Supplementary file 1 — ml4c00212_si_001.pdf [file ml4c00212_si_001.pdf]

Supplementary information:

Galectin-8N-Selective 4-Halophenyl-Phthalazinone–Galactals Double π-Stack in a Unique Pocket

Sjors van Klaveren,[1,2] Mujtaba Hassan,[1,2] Maria Håkansson,[3] Richard E. Johnsson,[4] Jessica Larsson,[4] Žiga Jakopin,[1] Marko Anderluh,[1] Hakon Leffler,[5] Tihomir Tomašič,[1,*] and Ulf J. Nilsson[2,*]

[1]Chair of Pharmaceutical Chemistry, University of Ljubljana, Faculty of Pharmacy, Aškerčeva cesta 7, 1000 Ljubljana, Slovenia
[2]Department of Chemistry, Lund University, Faculty of Science, Naturvetarvägen 14, 223 62, Lund, Sweden
[3]SARomics Biostructures AB, Medicon Village, SE-223 81, Lund, Sweden
[4]Red Glead Discovery AB, Medicon Village, SE-223 81, Lund, Sweden
[5]Department of Laboratory Medicine, Section MIG, Lund University, BMC-C1228b, Klinikgatan 28, 221 84, Lund, Sweden
*To whom correspondence should be addressed: Tihomir.Tomasic@ffa.uni-lj.si, ulf.nilsson@chem.lu.se

Table of contents

Safety statement S1

Comparative affinity analysis – galectin-8N (Met56) / Galectin-8N (Val56) S1

Table S1. Comparative fluorescence polarisation S1

Crystallisation, X-ray data collection and atomic structure determination S2

Table S2. X-ray crystallography data collection and refinement statistics S2

Superpositions of the X-ray structure with the two poses previously predicted by MD simulation S3

Figures S1-S2 S3

Physicochemical and *in vitro* ADME analyses S4

Table S3. Structures of tested compounds S4

Table S4. Solubility of compounds in 1% DMSO in phosphate buffer pH 7.4 S5

LogD$_{octanol/water}$ S5

Table S5. LogD of compounds in phosphate buffer(10 mM, pH 7.4) and 1-octanol S5

Chemical stability S6

Table S6. Chemical stability of compounds in acidic (pH 4) and basic (pH 9.5) condition S6

Plasma Stability S6

Table S7. Plasma stability of compounds at 1 µM over 8 hours. S7

Microsomal stability S7

Table S8. Liver microsomal stability (mouse) of compounds at 1 µM over 45 minutes. S8

Table S9. Liver microsomal stability (human) of compounds at 1 µM over 45 minutes S8

Parallel Artificial Membrane Permeability Assay (PAMPA) S9

Table S10. Permeability in PAMPA. S9

Figure S3. Caco-2/PAMPA correlation plot S10

Chromatography Instrumentation S11

General procedures, synthesis, data, and copies of NMR Spectra and Chromatograms S12

References S18

Safety statement: No unexpected or unusually high safety hazards were encountered
Comparative affinity analysis – galectin-8N (Met56) / Galectin-8N (Val56)

The two variants, galectin-8N (Met56) and galectin-8N (Val56), were procured from the Lund Protein Production Platform (LP3 - Lund, Sweden).

Human Galectin-8N 4–158-Met was cloned as amino acid 4–158 from human galectin-8 (UniProt entry O00214-1) without fusion or tags into pET26b(+), and the plasmid was transformed into *Escherichia coli* TUNER(DE3). LB medium, 8 L, supplemented with 50 µg/mL kanamycin, was inoculated to OD_{600} = 0.1 with an overnight culture of *E. coli* TUNER(DE3) / pET26b(+)_hGal8N_4-158-M. The culture was grown at 25°C, 250 rpm, 1 L / flask. At OD_{600} = 0.46, the temperature was lowered to 18°C. At OD_{600} = 0.9, IPTG was added to a final concentration of 1 mM. 20 hours after induction, the cells were harvested by centrifugation, resuspended in MEPBS, supplemented with three tablets of Complete Protease Inhibitor, EDTA-free (Roche) and a pinch of DNase I. The cell suspension (~100 mL) was passed two times through a French Pressure Cell at 18000 psi. The resulting lysate was ultracentrifuged, the supernatant passed through a 0.45 µm syringe filter, and then used for affinity chromatography. The 19 mL lactocyl-sepharose column was pre-equilibrated with 5 CV MEPBS before applying the sample. The run was performed with a flow rate of 2 mL/min at RT. The column was then washed with 10 CV MEPBS, and bound protein was eluted with 5 CV MEPBS+150 mM lactose. At 6 °C, 5 mL fractions were collected during elution. Pure elution fractions were pooled, concentrated, dialysed and further concentrated to give 13.1 mg/mL in 135 mL (1768 mg). The purity was >95%, estimated using SDS-PAGE analysis.

Human Galectin-8N 4–158-Val was cloned as amino acid 4–158 from human galectin-8 (56Val, UniProt entry O00214-1:p.Met56Val) without fusion or tags into pET26b(+), and the plasmid was transformed into *Escherichia coli* TUNER(DE3). LB medium, 9 L, supplemented with 50 µg/mL kanamycin, was inoculated to OD_{600} = 0.1 with an overnight culture of E. coli TUNER(DE3) / pET26b(+)_hGal8N_4-158-V. The culture was grown at 25°C, 250 rpm, 1 L / flask. At OD_{600} = 0.35, the temperature was lowered to 18°C. At OD_{600} = 0.86, IPTG was added to a final concentration of 0.1 mM. 20 hours after induction, the cells were harvested by centrifugation. The following preparation and chromatography were identical to that of galectin-8N (56Met), described above. Pure elution fractions were pooled, dialysed, and concentrated to give 4.4 mg/mL in about 100 mL (453 mg). The purity was >95%, estimated using SDS-PAGE analysis.

Affinities – galectin-8N (Met56) / Galectin-8N (Val56)
Compounds **1**, **2**, **6**, **7**, **9**, and **10** were evaluated for their affinity against galectin-8N Met56 to compare with the Val56 variant (Table S1).

Table S1. Comparative fluorescence polarisation. K_d (µM ±SEM)[a]

Compound	Val56	Met56	Structure:
**1**	82 ± 1.7	86 ± 5.2	Galactal–phthalazinone
**2**	78 ± 2.1	74 ± 1.2	Galactal–phthalazinone-4-phenyl
**6**	83 ± 1.9	73 ±1.7	Galactal–phthalazinone-4-phenyl-4-F
**7**	47 ± 4.7	39 ± 1.7	Galactal–phthalazinone-4-phenyl-4-Cl
**9**	43 ± 1.1	36 ± 1.7	Galactal–phthalazinone-4-phenyl-4-Me
**10**	50 ± 5.9	34 ± 3.2	Galactal–phthalazinone-4-phenyl-4-Br

[a] If not stated otherwise, results represent the mean ± SEM of n = 4 to 8 globally analysed from two independent experiments.

Crystallisation, X-ray data collection and atomic structure determination

Human galectin-8N was co-crystallised with lactose as described previously.[1] In brief: 12 mg/ml galectin-8N in 10 mM lactose, 10 mM Tris/HCl pH 8.0, 1 mM TCEP and 150 mM sodium chloride was mixed with 25 % (w/v) PEG 2000 monomethylether (PEG 2000 MME) and seed solution from crystals grown at the same condition (1.5 µL protein + 1.25 µL reservoir + 0.25 µL seed solution in a hanging drop over 1 ml reservoir using a NEXTAL plate). The experiment was setup at 295 K and crystals appeared within a few days. The co-crystals with lactose were used to soak in compound **10** by transferring crystals in three steps to different soaking drops. These were 2 µL drops with a soaking solution of 20% ethylene glycol, 25% PEG 2000 MME, 10 mM Tris pH 8, 1 mM TCEP, 50 mM NaCl, and 2 mM compound **10**. The incubation times in the first two drops were about 10 min and in the third drop about 24 h. Then the crystals were transferred to a 2 µL drop with the same constituents and flash-frozen in liquid nitrogen.

Data were collected at Diamond Light Source beamline I24 to 1.30 Å and processed using the Xia2 pipeline[2] and Aimless.[3] The structure was determined with two molecules in the asymmetric unit in space group $P2_12_12_1$. The structure has been refined starting from the 1.35 Å model of galectin-8N with lactose (PDB id: 7ALS) determined in the same space group. Anisotropic B-factor refinement were made using Refmac5,[4] the model building was made in Coot,[5] and the models have been analysed using Molprobity.[6]

Table S2. X-ray crystallography data collection and refinement statistics.

Data collection	Compound **10**, PDB ID: 9FXZ
Space group	$P2_12_12_1$
Cell dimensions<br>a, b, c (Å)	54.71, 62.28, 84.75
α, β, γ (°)	90, 90, 90
Wavelength (Å)	0.99990
Resolution (Å)	46.01 – 1.30<br>(1.32 – 1.30)
R_{merge}	0.055 (1.548)
Mean I/σ(I)	12.5 (1.1)
CC(1/2)	0.998 (0.593)
Completeness (%)	99.8 (99.9)
Redundancy	6.2 (5.4)
Refinement (Å)<br>Rwork/Rfree	46.01 – 1.30<br>0.157/ 0.200
No. of protein atoms in chain A, B	1232, 1224
No. of ligand atoms in chain A, B	29, 29
No. of solvent atoms	289
R.m.s. deviation of bonds (Å)	0.013
R.m.s. deviation of angles (°)	1.8
Ramachandran**<br>Favoured regions (%)<br>Allowed regions (%)<br>Outliers (%)	 99.3<br>0.7<br>0

Superpositions of the X-ray structure with the two poses previously predicted by MD simulation.

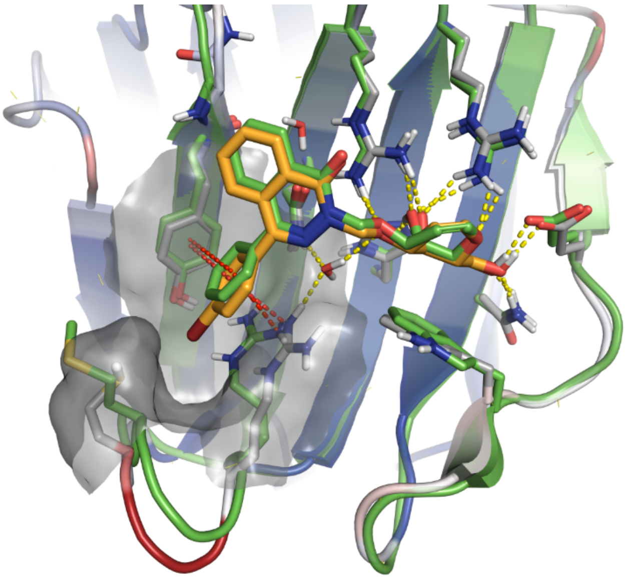

Figure S1 Superposition. Crystal structure of galectin 8N—galactal–phthalazinone **10** (PDB ID: 9FXZ) with the ligand presented in orange sticks, protein coloured in red-white-blue according to B-factor. In superposition, the molecular dynamics snapshot of initial binding mode of the 4-phenyl-phthalazinone **2** (published previously)[7] with the ligand and the protein presented in green sticks. Hydrogen bonding network is presented with yellow dotted lines, cation–π and π–π stacking are presented with red dotted lines. (Figure was generated with PyMOL v1.7, Schrodinger LLC.)

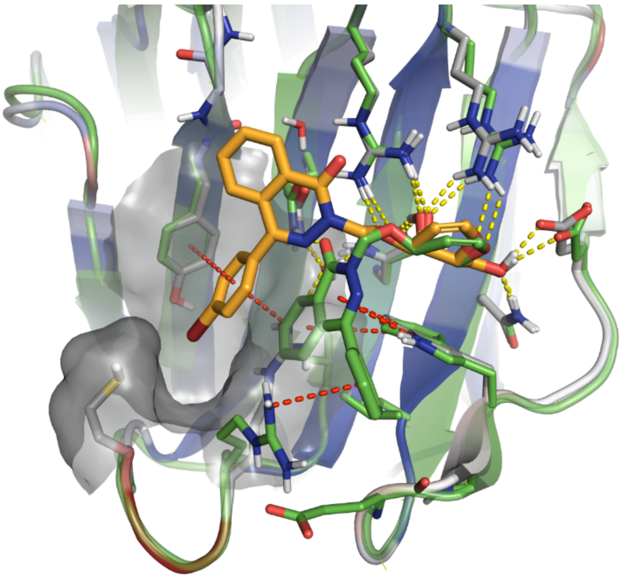

Figure S2 Superposition. Crystal structure of galectin 8N—galactal–phthalazinone **10** (PDB ID: 9FXZ) with the ligand presented in orange sticks, protein coloured in red-white-blue according to B-factor. In superposition, the molecular dynamics snapshot of an alternative binding mode of the 4-phenyl-phthalazinone **2** (published previously)[7] with the ligand and the protein presented in green sticks. Hydrogen bonding network is presented with yellow dotted lines, cation–π and π–π stacking are presented with red dotted lines. (Figure was generated with PyMOL v1.7, Schrodinger LLC.)

Physicochemical and *in vitro* ADME analyses

Table S3. Structures of tested compounds.

Compound	Structure
**1**[7]	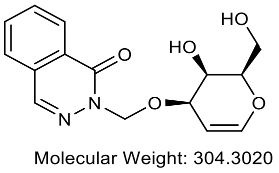
**2**[7]	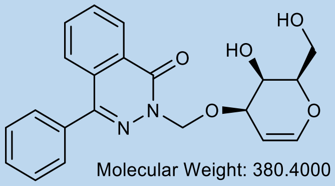
**6**	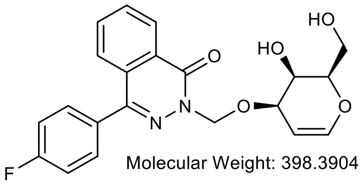
**7**	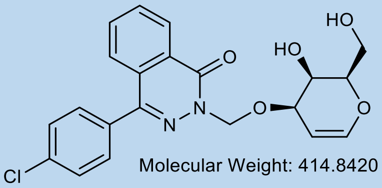
**9**	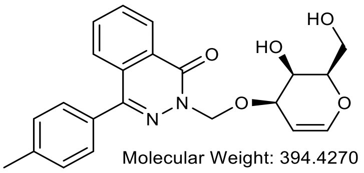
**10**	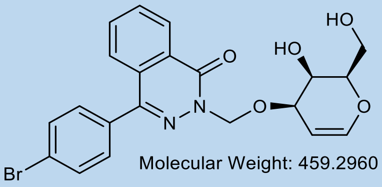

Screen solubility method

The starting materials for the assay were dissolved in 10 mM DMSO solutions. The solubility was assessed by dilution in Phosphate-buffer saline (PBS, 10 mM pH 7.4) to a concentration of 100 µM. The samples were equilibrated for 20−24 h, filtered (Millex LH 0.45 µM), and the solubility was determined by LC-UV in the reported range of 1−95 µM.

Table S4. Solubility of compounds in 1% DMSO in phosphate buffer pH 7.4

Compound	Results (µM)
**1**	>95
**2**	>95
**6**	94
**7**	63
**9**	>95
**10**	42

UV detection at 254 nm. Linear compound concentration–UV activity is presumed.

Experimental details

- Compound stock solutions (6 µL, 10 mM, prepared no more than 24 days prior)
- Dissolved in PBS (594 µL, 10 mM, pH 7.4)
- Samples were equilibrated overnight (24 h, StirStix, Alligator Magnetic Tumble stirrer, RT).
- Samples were LC-prepped with a syringe filter (Millex LH 0.45 µm)
- LC-UV quantification of filtered sample against a 1-point calibration curve.

LogD$_{octanol/water}$ pH 7.4

Distribution coefficients between 1-octanol and 10 mM phosphate buffer pH 7.4 were determined with a shake flask method with LC–UV detection of the compound concentration in both phases.

Compound stock solutions were added to 600 µL 1-octanol. Immediately, 600 µL of buffer were added to each vial. Vortex mixing was performed after addition of buffer. The equilibrium between the bi-phasic system were performed during 10-20 minutes at room temperature in a Thermomixer at 500 rpm. The two phases were separated by centrifugation. Prior to LC-UV analysis, the octanol phase was diluted 10 times in methanol in a new vial. The LC sample from the buffer phase were directly taken from the bottom layer by the autosampler.

Table S5 shows the obtained peak area and the calculated LogD for each compound. The LogD values for controls (cyclobenzaprine, glyburide) were within reference values. logD = log {[compound area in octanol]*10*50/[compound area in buffer]*5}.

Table S5. LogD of compounds in phosphate buffer(10 mM, pH 7.4) and 1-octanol.

Compound	Ret. Time Buffer	Peak area Buffer	Ret. Time Octanol	Peak area Octanol	logD
Cyclobenzaprine	5.05	10.60	5.05	62.46	2.8
Glyburide	5.81	19.80	5.75	27.00	2.1
**1**	3.56	175.90	3.49	16.23	1.0
**2**	4.85	92.30	4.78	44.40	1.7
**6**	4.94	102.11	4.87	52.79	1.7
**7**	5.27	34.47	5.18	74.05	2.3
**9**	5.38	25.37	5.25	85.61	2.5
**10**	5.19	38.36	5.10	63.76	2.2

UV detection at 254 nm. Linear compound concentration–UV activity is presumed. Reference values: cyclobenzaprine 2.6 ± 0.3, glyburide 2.1 ± 0.3.

Experimental details

- Compound stock solutions (6 µL, 10 mM, prepared no more than 24 days prior)
- Phosphate buffer (600 µL, 10 mM, pH 7.4)
- 1-Octanol (600 µL)
- Samples were mixed in a thermomixer (10 min, 500 rpm, room temp.)
- Layers were separated by centrifugation (1000 G, 6 min)
- 25 µL of supernatant octanol was transferred to vials containing 225 µL methanol

LC-UV chromatograms were obtained for both phases, different injection volumes were used to increase the reported range.

Chemical stability

Chemical stability was determined by monitoring the diminishing of the parent compound over several days in acidic and basic media. The stability was measured based on the UV response of the compound in LC-UV analysis, which diminishes with the formation of degradation products. Compound stock solutions were diluted in ammonium acetate buffers (pH 4 or pH 9.5) to a concentration up to 100 µM with 1% DMSO. The first measurement was taken immediately after dissolution of the compound. The samples were mixed in a rolling mixer for 5 days, and the samples were analysed at 3 time points (0 h, 24 h, and 120 h). **Table S6** shows the remaining percentages of the parent compounds present after 5 days of agitation.

Table S6. Chemical stability of compounds in acidic (pH 4) and basic (pH 9.5) conditions.

Compound	% remaining (pH 4, Day 5)	% remaining (pH 9.5, Day 5)
**1**	96%	> 99%
**2**	> 99%	> 99%
**6**	97%	> 99%
**7**	97%	99%
**9**	96%	> 99%
**10**	91%	95%

UV detection at 254 nm. Results over 95% are considered ‘stable’. UV activity and solubility of degradation products is presumed.

Experimental details

- Compound stock solutions (2 x 6 µL, 10 mM, prepared no more than 24 days prior)
- Dissolved in Ammonium acetate buffer (594 µL, pH 4)
- Dissolved in Ammonium acetate buffer (594 µL, pH 9.5)
- Samples were prepared for LC with a syringe filter (Millex LH 0.45 µm)
- Samples were mixed in a gentle rolling mixer (RM-810) for 5 days.
- Area% obtained by LC-UV directly from the sample.

Plasma Stability

In vitro Plasma Stability Assay.

The reaction was started by the addition of the compound to blank plasma in LC vials after prewarming of the plasma at 37 °C. The initial compound concentration was 1 µM. The reaction was stopped by pipetting a sample to a new vial containing acetonitrile at seven time points (0, 0.17, 0.5, 1, 2, 4, and 8 h). Time point 0 h was about 15−20 s after spiking the plasma with the compound. The precipitated samples were centrifuged, and the supernatant were diluted and analysed by LC−MS/MS to determine the % of compound remaining.

Table S7 shows the remaining percentages of compound at each time-point and the half-life of the compounds determined by linear regression of the natural logarithm of the concentration percent over time. T½ = ln2/-k.

Table S7. Plasma stability of compounds at 1 µM over 8 hours.

Compound	0 h	0.17 h	0.5 h	1 h	2 h	4 h	8 h	Half-life (h)
**1**	100	-[a]	97	90	86	89	88	>16
**2**	100	>100	>100	>100	>100	>100	>100	>16
**6**	100	100	98	88	81	88	86	>16
**7**	100	99	95	97	95	96	90	>16
**9**	100	89	89	88	83	65	-[a]	7.6
**10**	100	95	90	91	81	84	85	>16

Values are relative to 0 h timepoint. Peak area derived from LC-MS/MS MRM chromatogram. [a] Outliers (> 3x SD) were removed.

Experimental details

- Compound stock solutions (3 µL, 10 mM, prepared no more than 24 days prior)
- Spike solution: Ethanol/H_2O (1:3, 297 µL)
- 'stop'-solution: acetonitrile (180 µL)
- Pooled human blood plasma (Innovative Research, Inc., IPLAK2E100ML-32215)*
- Plasma (594 µL per experiment) was tempered to 37 °C on a thermomixer
- Compound sample solution (6 µL, 100 µM) was spiked into the respective plasma containers
- Mixture was vortexed and the first (0 h) sample (60 µL) transferred to a 'stop'-vial
- At each time-point, the mixture was vortexed and a sample (60 µL) transferred to a 'stop'-vial
- LC-MS/MS detection by MRM scan.

Pooled human blood plasma (Innovative Research, Inc., IPLAK2E100ML-32215) was collected from donors via apheresis, aliquoted and shipped, and stored frozen. Each unit is tested for viral markers and found negative for HBsAg, HCV, HIV-1, HIV-2, HIV-1Ag or HIV-1 NAT, ALT, and syphilis using FDA-approved methods. To the NA Citrate-collected product was added an anticoagulant K2 EDTA. Plasma was used within one year of collection, well within the industry standard expiration of 3 years from Date of Manufacture. Stored at -20 °C.

Microsomal stability

Metabolic Stability in Liver Microsomes.

The assay was run in a 96-deep well format and solutions were prepared containing microsomes in phosphate buffer. The microsomes employed were from humans and mice. The reaction was initiated by addition of NADPH after preincubation at 37 °C with test compounds. The reaction was stopped by the addition of acetonitrile (MeCN) at six time points (0, 5, 10, 15, 25, and 45 min), the samples were centrifuged, and the supernatant was diluted to determine the loss of compound by LC−MS/MS. Two incubations per compound per species. For further details regarding working concentrations, volumes, and final concentrations at incubation, see below.

Table S8 (mouse) and **Table S9 (human)** show the intrinsic clearance (CL_{int})of each compound determined by linear regression of the initial disappearance, and the associated half-life of the compounds. The half-lives obtained in this method are depending on the microsomal concentration.

Table S8. Liver microsomal stability (mouse) of compounds at 1 µM over 45 minutes.

Compound	CL_{int} (µL/min/mg protein)		CL_{int} Average (µL/min/mg protein)	Half-life (min)
Diphenhydramine	52		52	21
Formoterol	16		16	107
**1**	<10	<10	<10	>140
**2**	78	91	85	17
**6**	50	51	51	27
**7**	56	65	61	23
**9**	136	138	137	10
**10**	38	39	39	36

CL_{int} determined by linear regression of peak area derived from LC-MS/MS chromatogram of samples at each time point. Reference clearance values: diphenhydramine 64 ± 20, formoterol 26 ± 10.

Table S9. Liver microsomal stability (human) of compounds at 1 µM over 45 minutes.

Compound	CL_{int} (µL/min/mg)		CL_{int} Average (µL/min/mg)	Half-life (min)
Dextromethorphan	13		13	107
Verapamil	141		141	10
**1**	<10	<10	<10	>140
**2**	<10	<10	<10	>140
**6**	<10	<10	<10	>140
**7**	12	<10	10	-
**9**	<10	<10	<10	>140
**10**	11	11	11	126

CL_{int} determined by linear regression of peak area derived from LC-MS/MS chromatogram of samples at each time point. Reference clearance values: dextromethorphan 25 ± 10, verapamil 150 ± 25.

Experimental details

- Compound stock solutions (3 µL, 10 mM, prepared no more than 24 days prior)
- Dissolved in acetonitrile (297 µL)
- Na_4NAPDH solution (7.5 mM) in phosphate buffer (1.9 mL)
- 'stop'-solution: acetonitrile (80 µL per timepoint)
- Liver microsomal protein (GIBCO mouse CD1 microsomes 20 mg/mL Cat. Nr. MSMCPL or GIBCO pooled human microsomes 20mg/mL Cat. Nr. HMMCPL, Fischer Scientific)
- Microsome suspension: 0.577 mg/mL in phosphate buffer(0.1 M, pH 7.4)
- Microsome suspension (520 µL per compound) was tempered to 37 °C on a thermomixer
- Compound sample solution (6 µL, 100 µM) was spiked into the microsome suspensions
- Equilibrated for 5 minutes
- NADPH solution (tempered, 80 µL) added to initiate microsomal activity
- Suspensions were mixed and the first (0 min) sample (80 µL) transferred to the 'stop'-plate
- At each time-point, after mixing, a sample (80 µL) was transferred to the 'stop'-plate
- 'stop'-plate centrifuged (2000 G, 10 min, 18 °C)
- Supernatant 'stop'-solution (60 µL) transferred to analysis plate (water, 120 µL)
- LC-MS/MS analysis by MRM scan.

Parallel Artificial Membrane Permeability Assay (PAMPA)

The membrane permeability is determined using a pre-coated tri-layer PAMPA Plate System. The plate system is made up of a donor- and an acceptor-compartment separated by a porous filter coated with a lipid-oil-lipid tri-layer. Compound stock solutions were diluted in PBS (10 mM, pH 7.4) / 10% methanol to a concentration up to 100 µM with 1% DMSO. This compound solution was added to the donor-compartment, and the same solvent mixture without compound was added to the acceptor-compartment. After 5 hours of equilibration, the compound concentration in both the donor- and acceptor-compartment were compared based on the peak area obtained by LC-UV analysis. **Table S10** shows the membrane permeability calculated from the average of four replicates. Because PAMPA is an artificial permeability assay, results must be interpreted by comparison to *in vitro* Caco-2 data; the relative permeability of known and tested compounds is a useful indicator of *in vitro* and *in vivo* permeability.

Table S10. Permeability (values ± SD) of compounds up to 100 µM in PBS (10 mM, pH 7.4) / 10% methanol in a PAMPA.

Compound	Permeability (10-6 cm/s)	Tri-layer PAMPA[8] (10-6 cm/s)	Caco-2[9] (10-6 cm/s)
Antipyrine	**8.9** ± 0.85	8.42 ± 0.32	28.8 ± 3.5
Caffeine	10.4 ± 1.69	9.58 ± 0.63	33.1 ± 2.7
Naproxen	**4.4** ± 1.31	6.03 ± 0.59	30 ± 4.1
Amiloride HCl	**0.0** ± 0.10	0.08 ± 0.02	4.9 ± 0.8
Dexamethasone	**1.3** ± 0.49	2.71 ± 0.41	13.4 ±1.3
Nadolol	**0.0** ± 0.00	0.0 ± 0.0	1.7 ± 0.4
**1**	**1.9** ± 0.35		
**2**	**5.4** ± 0.88		
**6**	**6.7** ± 1.55		
**10**	**7.4** ± 1.50		

UV detection at 254 nm. Linear compound concentration–UV activity is presumed.

Experimental details

- Corning® BioCoat™ Pre-coated PAMPA Plate System (Cat. No. 353015).
- Donor solution
  - Compound stock solutions (15 µL, 10 mM, prepared no more than 24 days prior)
  - Phosphate buffer (1335 µL, 10 mM, pH 7.4)
  - Methanol (150 µL)
  - Mixed and transferred with a syringe filter (Millex LH 0.45 µm)
  - 300 µL per donor well.
- Acceptor solution
  - Phosphate-buffered saline (1080 µL, 10 mM, pH 7.4)
  - Methanol (120 µL)
  - Mixed and transferred with a syringe filter (Millex LH 0.45 µm)
  - 200 µL per acceptor well.
- Equilibration for 5 hours.
- Donor and acceptor solutions transferred to separate deep-well plates.
- LC-UV quantification.

Calculation

The apparent permeability is calculated as follows:

$P_{app} = (-\ln(1-C_A/C_{Eq}))/(A*t*(1/V_D+1/V_A)$

In which the equilibrium concentration (C_{Eq}) is determined through:

$C_{Eq} = (C_D*V_D+C_A*V_A)/(V_D+V_A)$

Constants:

V_D	Donor well volume	0.3 mL
V_A	Acceptor well volume	0.2 mL
t	incubation time	18000 s
A	Filter area	0.3 cm^2

Variables:

C_D	Concentration in donor-compartment
C_A	Concentration in acceptor-compartment

Linear compound concentration–UV activity is presumed; the compound peak area in the LC-UV analysis replaces C_D and C_A in the formulae.

Short assay validation

This PAMPA was validated by the inclusion of 6 reference compounds (**Table S8**) and comparison to the literature provided by the assay vendor.[8] The UV data obtained in the experiment is close to the reference data for the Tri-layer PAMPA, which was also determined by UV. The predictive permeability determined by PAMPA is only valuable in its correlation to Caco-2 data (**Figure S3**). We found that the testa with 10% MeOH (to increase compound solubility) gave the most accurate results compared to the references (see the R^2 in the plotted data below), so these should be regarded.

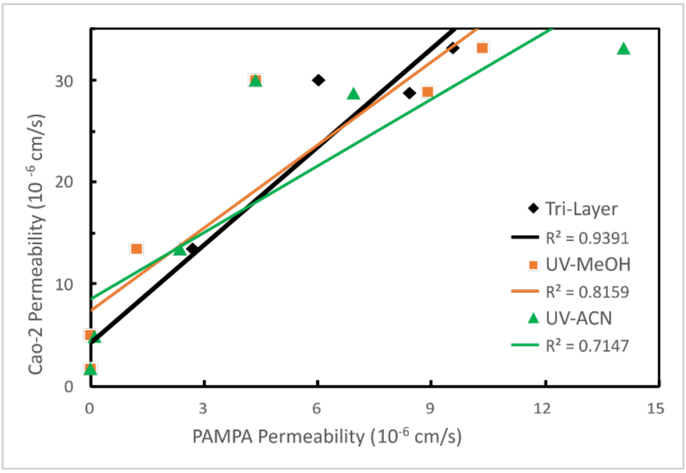

Figure S3. Caco-2/PAMPA correlation plot of the PAMPA data of the reference compounds presented in **Table S8** is set out against Caco-2 data,[9] and compared to the reference performance of the tri-layer PAMPA.[8]

Chromatography Instrumentation

**LC-MS/MS analysis**	(Plasma Stability, Microsomal stability)
Autosampler	Shimadzu NexeraXR SIL-20AC
Degasser	Shimadzu FCV-11AL
Gradient LC-pumps	Two Shimadzu NexeraXR LC-20AD pumps
Oven	Shimadzu CTO-20AC
System controller	Shimadzu CBM-20A
Mass spectrometer	Sciex QTRAP4500 with Analyst software 1.6.3
MS-probe	Turbospray ESI+

Standard analytical parameters

Analytical Flow	0.3 mL/min
Analytical column	Phenomenex LUNA Omega C18 Polar 100Å, 1.6 µm, column dim: 50x2.1 mm
Injection volume	10 µL
Column temperature	+40°C
Divert valve	1.2 minutes
Mobile phase A	H_2O/MeCN/Formic acid 95/5/0.1
Mobile phase B	H_2O/MeCN/Formic acid 5/95/0.1
MS detection	MRM; ESI Positive mode

**LC-UV analysis**	(Solubility, Chemical stability, LogD, PAMPA)
Autosampler	Agilent 1260 Multisampler, G7167A
Gradient LC-pumps	Agilent 1260 Flexible Pump, G7104C
UV-detector	Agilent 1260 DAD HS, G7117C
Column oven	Agilent 1260 MCT, G7116A
Mass spectrometer	Agilent InfinityLab MSD iQ, G6160A

Standard analytical parameters

Analytical Flow	0.5 mL/min
Analytical column	Luna Omega 1.6 µm PS C18 100 Å, column dimensions: 50 x 2.1 mm
Injection volume	5 µL
Mobile phase A	H_2O/MeCN/Formic acid 95/5/0.1
Mobile phase B	MeCN/Formic acid 100/0.1
Column temp	35°C
UV-detection	254 nm

Materials

Vials	LC glass vials, for snap caps, 2mL, Scantec
	LC glass vials, µ Vial i2V, Verex, Phenomenex
	Snap Caps, PTFE/silicone septa, Verex, Phenomenex
	LC glass vials, screw, Agilent Technologies
	Screw caps, PTFE/silicone septa, Agilent technologies
Pipettes	Single or multi-channel automatic pipettes, Finnpipette with suitable Finntips
Eppendorf tubes	1.5 mL, Sarstedts

Solvents

Acetonitrile (MeCN)	LiChrosolv, hypergrade for LC-MS, Merck Chemicals
Ethanol	Solveco, 99.7% Absolute, Spectrographic
Formic acid (HCOOH)	For LC-MS, Carlo-Erba
Water	> 18MΩcm quality (Milli-Q, Millipore)
PBS	Medicago, PBS tablets, Lot 190716

General synthesis procedures, synthesis experiments, and data

Chemistry

All reagents and solvents were dried before use. Commercial reagents were used without further purification. TLC analysis was performed on precoated Merck silica gel 60 F254 aluminium plates using UV light and charring solution (H_2SO_4: EtOH 1: 9) with heating. Flash column chromatography was performed on SiO_2 purchased from Aldrich (technical grade, 60 Å pore size, 230–400 mesh, 40–63 µm). Preparative HPLC was performed on an Agilent 1260 Infinity system with a SymmetryPrep C18, 5 µM, 19 mm × 100 mm column using a gradient (water with 0.1% formic acid and acetonitrile). Monitoring and collection were based on UV−vis absorbance at 210 and 254 nm. Automated reversed phase chromatography was performed on a Biotage® Isolera™ with a Sfär C18 D-Duo 100 Å 30 µm column column using a gradient (water and acetonitrile). Monitoring and collection were based on UV−vis absorbance at 254 nm. NMR spectra ^{1}H, ^{13}C, 2D COSY, and HMQC were recorded with a Bruker Avance II 400 MHz spectrometer (400 Hz for ^{1}H, 100 Hz for ^{13}C) or a Bruker Avance III 500 MHz spectrometer (500 Hz for ^{1}H, 125 Hz for ^{13}C) at ambient temperature. Chemical shifts are reported in δ parts per million (ppm), with multiplicity (b = broad, s = singlet, d = doublet, t = triplet, q = quartet, quin = quintet, hept = heptet, m = multiplet), coupling constants (in Hz) and integration. High-resolution mass analyses were performed using a Micromass Q-TOF mass spectrometer (ESI). Purities of final compounds were determined by UPLC (Waters Acquity UPLC system, column Waters Acquity CSHC$_{18}$, 0.5 mL min−1 H_2O–MeCN gradient 5–95% 10 min with 0.1% formic acid). Analytical data are given for tested compounds. All tested compounds were ≥95% pure according to analytical HPLC analysis.

General procedure A, hydrazine condensation[10]

Functionalised 2-(benzoyl)benzoic acid (1 equiv.) was dissolved (with approx. 5 mL/mmol) in ethanol. Hydrazine hydrate (3 equiv.) was added and the mixture was refluxed for 4 h. The mixture was allowed to cool, solids were filtered off, washed with cold EtOH, and dried *in vacuo* to give the product.

General procedure B1 and B2, alkylation with chloromethyl pivalate[7,11]

Phthalazinone (1 equiv.), chloromethyl pivalate (1.2 equiv.), and base were dissolved and heated to 70 °C for 1.5 h. For procedure B1: Cs_2CO_3 (1.5 equiv.) in dry DMF (with approx. 5 mL/mmol phthalazinone); for procedure B2: NaH (2 equiv. at start, and an additional 2 equiv. after 1 h) in dry THF (with approx. 15 mL/mmol phthalazinone). After the reaction, the solvent was evaporated in vacuo. The product was extracted (EtOAc/HCl_{aq} 0.5 M), organic layers were combined dried over Na_2SO_4, filtered, and concentrated *in vacuo* to give the product.

General procedure C, bromination with hydrogen bromide[7,11]

N-methyl pivalate (1 equiv.) was dissolved (with approx. 8 mL/mmol) in glacial acetic acid, and hydrogen bromide (1.5 equiv., 33% wt in acetic acid) was added. The mixture was heated to 75 °C for 2 h, and then allowed to cool to room temperature. The addition of ice precipitated the product, which was filtered off and dried *in vacuo*. Bromides that did not precipitate were diluted with water and the products extracted with EtOAc. The organic layers were combined, washed with brine, dried over Na_2SO_4, filtered, and concentrated *in vacuo* to give the products.

General procedure D, regioselective *O*-alkylation with dibutyltin oxide[7,12]

N-methyl halide (1 equiv.), D-galactal (1.1 equiv.), potassium carbonate (1.5 equiv.), dibutyltin oxide (1.2 equiv.), and tetrabutylammonium iodide (1.1 equiv.) were charged to a microwave vessel and suspended (with approx. 10–15 mL/mmol) in a mixture of acetonitrile and toluene (1:5). The mixture was agitated to 120 °C for 30 min by microwave irradiation. At room temperature, the mixture was filtered and dry loaded on silica for column chromatography (EtOAc/Heptane). Final purification of these final products was performed by automated reversed-phase column chromatography or by preparative HPLC.

Competitive fluorescence polarisation experiments

Human galectin-1, -3, -4C, -4N, -7, -8C, -9C, and -9N were expressed and purified as previously described.[13–15] Galectin-8N was expressed and purified as described in the supplementary information. Fluorescence polarisation experiments were performed using the PHERAstar FS plate reader (software version 2.10 R3), and the fluorescence anisotropy of fluorescein tagged probes were measured by excitation at 485 nm and emission at 520 nm. The specific conditions and probes used for galectin-1, -3, -4C, -4N, -7, -8C, -8N, -9C, and -9N were kept as reported.[13–15] In this assay a fixed concentration of galectin (e.g., 100 nM for Gal-8N) and trace concentration of a fluorescein-tagged probe (typically 4 nM) are mixed with a series of concentration of inhibitor to be tested. The measured anisotropy will represent an average of the value for free probe (typically around 30) and the value for 100% galectin bound probe (determined in a separate experiment), linearly weighted by their relative concentration. From this the free and bound probe concentration was calculated for each data point and, together with K_d for the galectin-probe interaction (from a separate experiment without inhibitor), the concentration of free galectin was calculated by the law of mass action. Inhibitor bound galectin concentration was calculated from total galectin concentration minus concentration of free galectin and the small amount bound to the probe, and then free inhibitor from total inhibitor minus galectin-bound inhibitor. Now with known concentrations of free inhibitor, galectin-inhibitor complex, and free galectin, the K_d of the galectin inhibitor interaction was calculated from law of mass action.[15] The synthesised compounds were dissolved in pure DMSO at 20 mM concentration and diluted in PBS to 3–6 different concentrations, and each concentration was tested in duplicate. The highest inhibitor concentrations tested were 1.5 mM. The average values of K_d and SEM were calculated from 4 to 8 duplicate measurements, showing 10–90% inhibition.

(4-(4-Fluorophenyl) phthalazin-1(2*H*)-one-2-ylmethyl) pivalate (4a)

The compound was prepared according to general procedure B1 starting from 4-(4-fluorophenyl)-phthalazin-1(2*H*)-one **3a** (150 mg, 0.62 mmol). Column chromatography (1:2 EtOAc/Hept.) gave intermediate **4a** as a white powder (180 mg, 81% yield). ^{1}H NMR (400 MHz, $CDCl_3$) δ 8.59 – 8.52 (m, 1H), 7.85 – 7.77 (m, 2H), 7.73 – 7.66 (m, 1H), 7.62 – 7.55 (m, 2H), 7.25 – 7.19 (m, 2H), 6.19 (s, 2H), 1.22 (s, 9H).

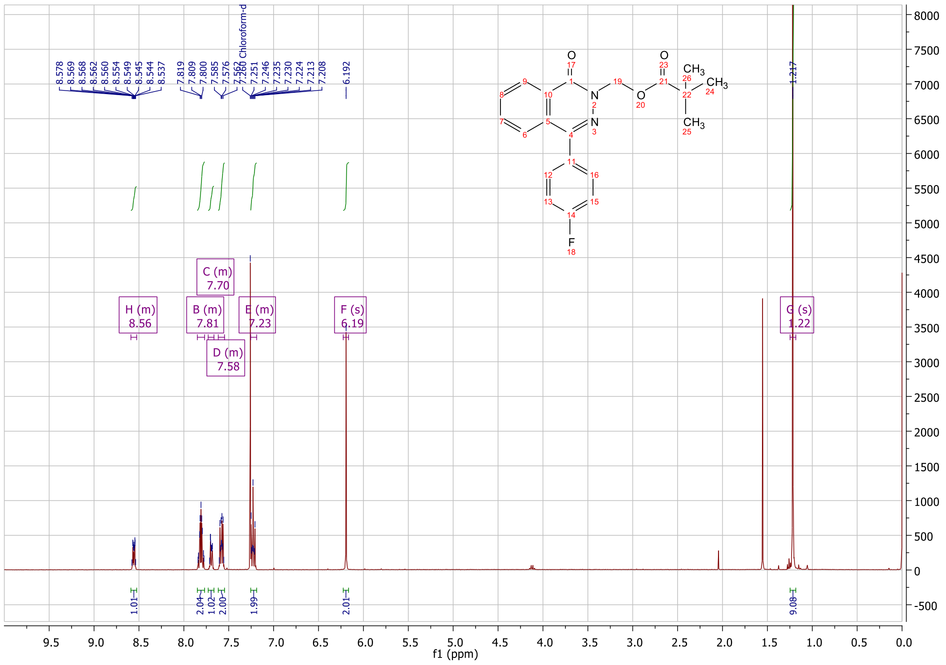

2-(Bromomethyl)-4-(4-fluorophenyl)phthalazin-1(2*H*)-one (5a)

The compound was prepared according to general procedure C using intermediate **4a** (100 mg, 0.28 mmol) to give intermediate **5a** as a white powder (79 mg, 84% yield). ^{1}H NMR (400 MHz, $CDCl_3$) δ 8.57 – 8.53 (m, 2H), 7.86 – 7.78 (m, 4H), 7.72 – 7.67 (m, 2H), 7.64 – 7.57 (m, 2H), 7.28 – 7.20 (m, 11H), 6.05 (s, 4H).

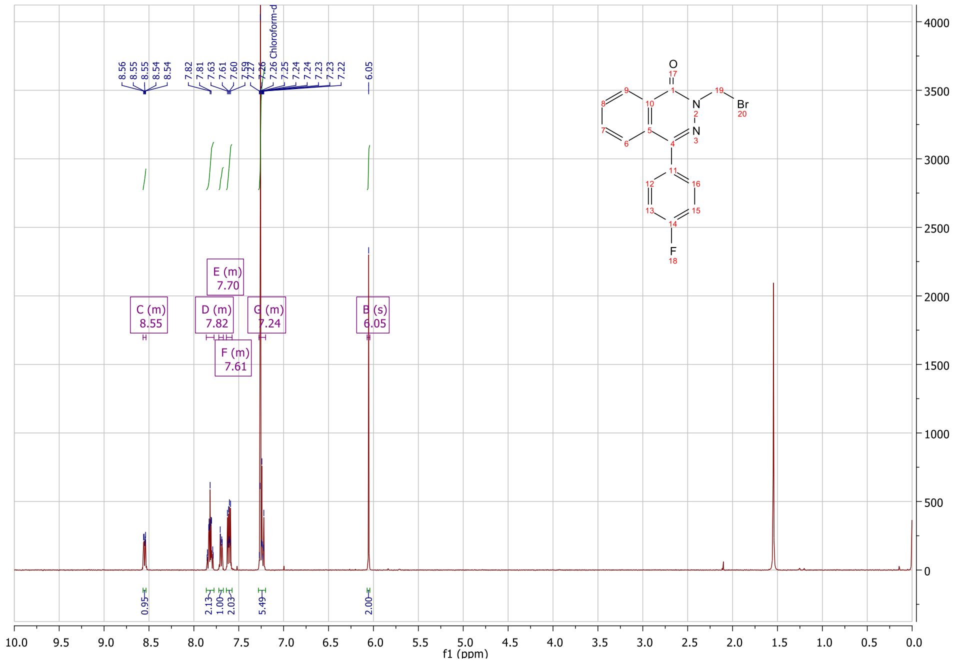

3-*O*-(4-(4-fluorophenyl)phthalazin-1(2*H*)-one-2-ylmethyl)-D-galactal (Compound 6)

^{1}H NMR (400 MHz, $CDCl_3$): δ 8.58 – 8.51 (m, 1H), 7.87 – 7.78 (m, 2H), 7.74 – 7.68 (m, 1H), 7.62 – 7.55 (m, 2H), 7.26 – 7.19 (m, 2H), 6.46 (dd, *J* = 6.3, 1.6 Hz, 1H), 5.81 (d, *J* = 9.3 Hz, 1H), 5.67 (d, *J* = 9.3 Hz, 1H), 4.72 (dt, *J* = 6.3, 2.0 Hz, 1H), 4.56 – 4.50 (m, 1H), 4.32 – 4.27 (m, 1H), 4.09 – 4.01 (m, 1H), 4.00 – 3.95 (m, 1H), 3.94 – 3.85 (m, 1H), 3.61 (d, *J* = 2.7 Hz, 1H), 2.47 (dd, *J* = 8.4, 3.0 Hz, 1H).

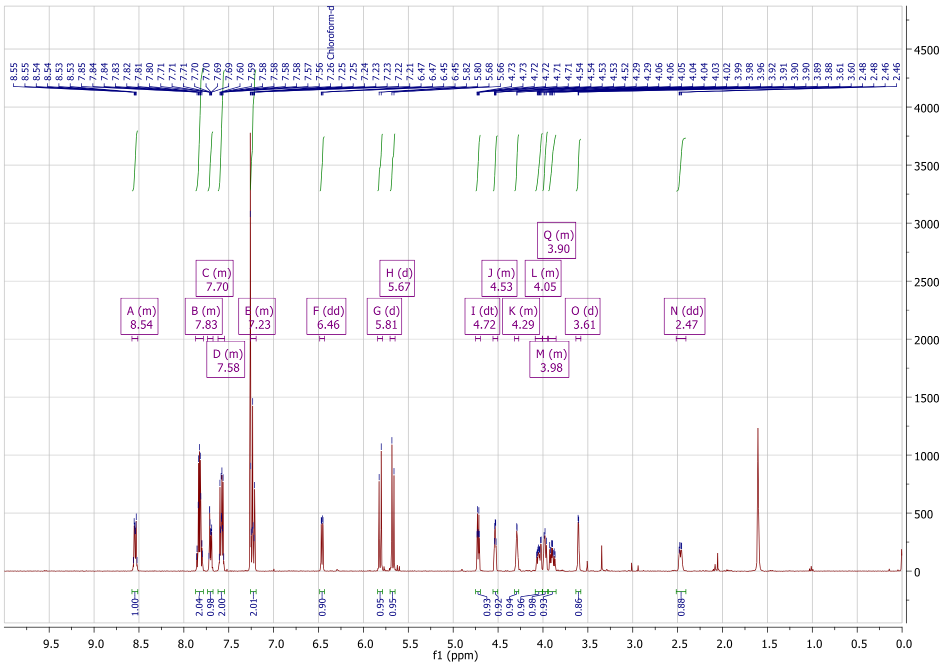

3-*O*-(4-(4-fluorophenyl)phthalazin-1(2*H*)-one-2-ylmethyl)-D-galactal (6)

The compound was prepared according to general procedure D using intermediate **5a** (40 mg, 0.139 mmol), D-galactal (21 mg, 0.144 mmol), TBAI (52 mg, 0.140 mmol), K_2CO_3 (30 mg, 0.217 mmol) and dibutyltin oxide (39 mg, 0.157 mmol). Column chromatography (1:40 MeOH/DCM), followed by reversed-phase automated column chromatography gave compound **6** as a white solid (22 mg, 40% yield). ^{1}H NMR (400 MHz, $CDCl_3$) δ 8.58 – 8.51 (m, 1H), 7.87 – 7.78 (m, 2H), 7.74 – 7.68 (m, 1H), 7.62 – 7.55 (m, 2H), 7.26 – 7.19 (m, 2H), 6.46 (dd, *J* = 6.3, 1.6 Hz, 1H), 5.81 (d, *J* = 9.3 Hz, 1H), 5.67 (d, *J* = 9.3 Hz, 1H), 4.72 (dt, *J* = 6.3, 2.0 Hz, 1H), 4.56 – 4.50 (m, 1H), 4.32 – 4.27 (m, 1H), 4.09 – 4.01 (m, 1H), 4.00 – 3.95 (m, 1H), 3.94 – 3.85 (m, 1H), 3.61 (d, *J* = 2.7 Hz, 1H), 2.47 (dd, *J* = 8.4, 3.0 Hz, 1H). ^{13}C NMR (101 MHz, $CDCl_3$) δ 163.59 (d, *J* = 249.7 Hz), 160.23, 147.46, 145.61, 133.91, 132.18, 131.48 (d, *J* = 8.4 Hz), 130.79 (d, *J* = 3.4 Hz), 129.58, 128.24, 127.74, 127.02, 116.01 (d, *J* = 21.8 Hz), 78.39, 77.16, 76.18, 70.68, 64.28, 63.16. HPLC purity: 95.1%, HRMS calcd for $C_{21}H_{20}O_5N_2F + H^+$ ($M + H^+$): 399.13508, found: 399.13552.

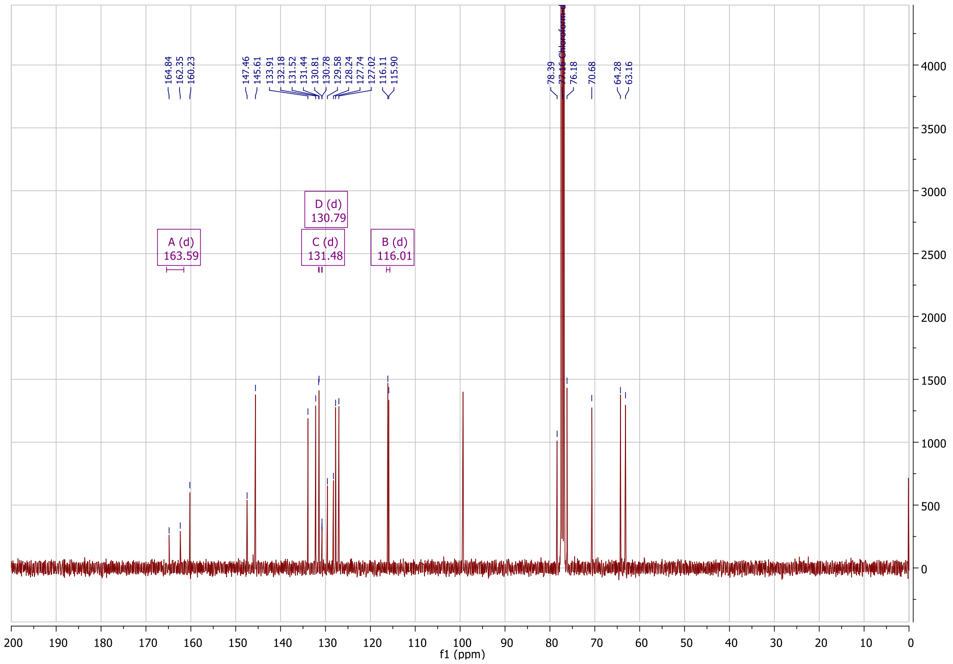

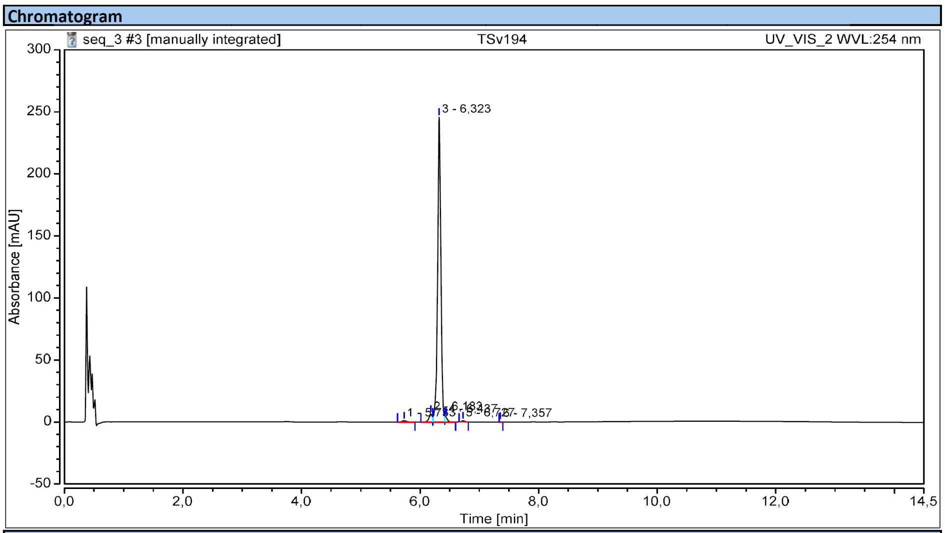

Integration Results

No.	Peak Name	Retention Time min	Area mAU*min	Height mAU	Relative Area %	Relative Height %	Amount n.a.
1		5,733	0,108	1,149	0,66	0,44	n.a.
2		6,183	0,410	6,360	2,51	2,45	n.a.
3		6,323	15,530	245,868	95,08	94,79	n.a.
4		6,437	0,211	4,221	1,29	1,63	n.a.
5		6,727	0,061	1,041	0,37	0,40	n.a.
6		7,357	0,013	0,751	0,08	0,29	n.a.
**Total:**			**16,333**	**259,391**	**100,00**	**100,00**	

4-(4-Chlorophenyl)phthalazin-1(2*H*)-one (3b)

2-(4-Chlorobenzoyl)benzoic acid (2.50 g, 9.59 mmol) was dissolved in ethanol (50 mL). Hydrazine hydrate (1.40 mL, 28.77 mmol) was added and the mixture was refluxed for 4 h. The mixture was allowed to cool, solids were filtered off, washed with EtOH, and dried *in vacuo* to give intermediate **3b** as a white powder (2.21 g, 90% yield). ^{1}H NMR (400 MHz, $CDCl_3$) δ 10.04 (s, 1H), 8.56 – 8.48 (m, 1H), 7.86 – 7.77 (m, 2H), 7.76 – 7.68 (m, 1H), 7.58 – 7.48 (m, 4H).

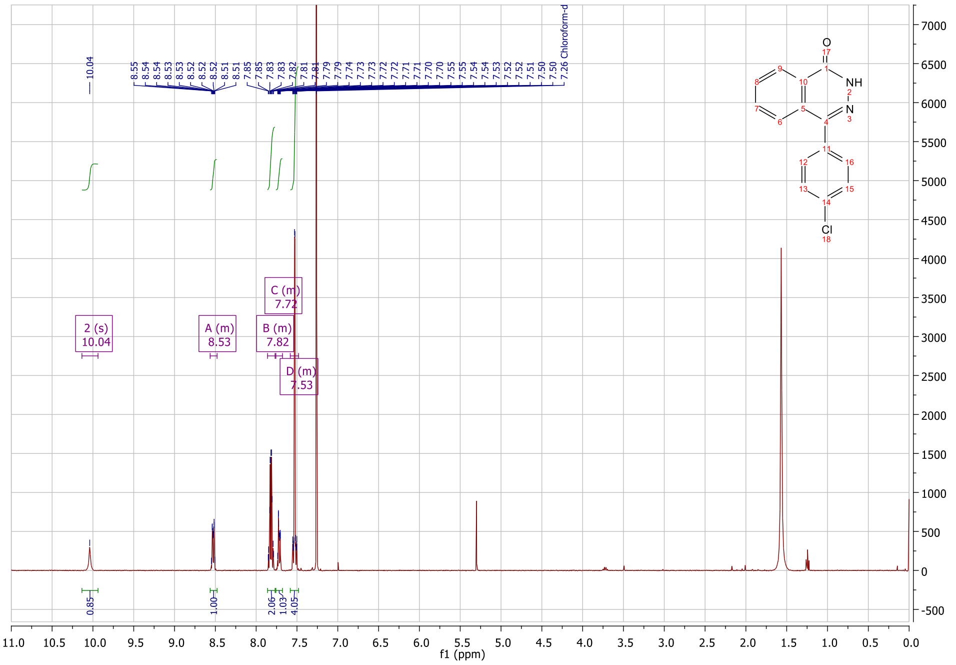

(4-(4-Chlorophenyl)phthalazin-1(2*H*)-one-2-yl)methyl pivalate (4b)

The compound was prepared according to general procedure B2 using intermediate **3b** (500 mg, 1.95 mmol) to give intermediate **4b** as a white powder (684 mg, 95% yield) ^{1}H NMR (400 MHz, $CDCl_3$) δ 8.58 – 8.52 (m, 1H), 7.86 – 7.77 (m, 2H), 7.72 – 7.66 (m, 1H), 7.57 – 7.49 (m, 4H), 6.19 (s, 2H), 1.21 (s, 9H).

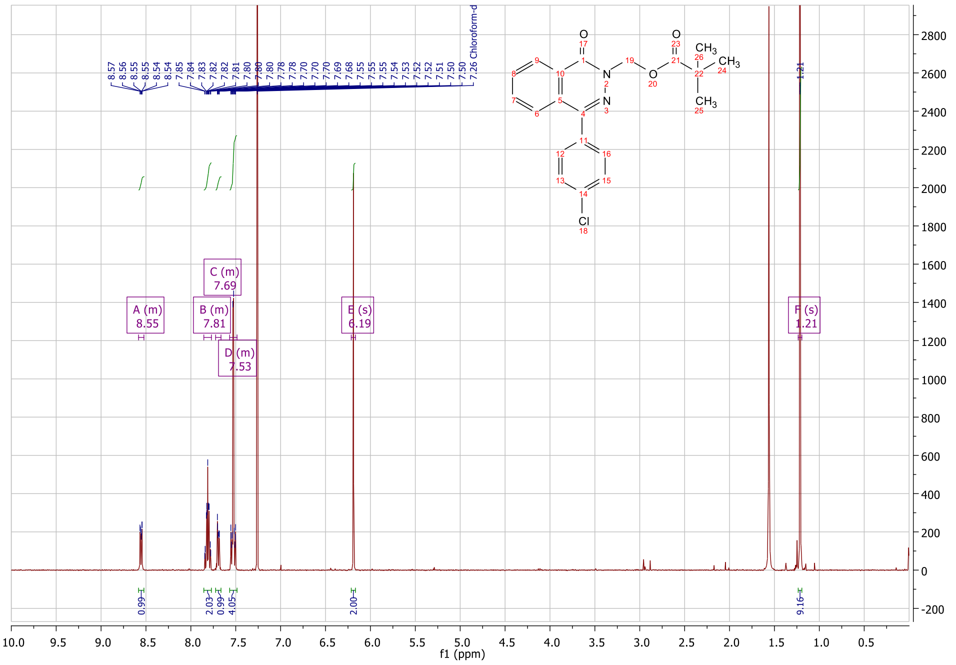

2-(Bromomethyl)-4-(4-chlorophenyl)phthalazin-1(2*H*)-one (5b)

The compound was prepared according to general procedure C using intermediate **4b** (680 mg, 1.83 mmol) to give intermediate **5b** as a white powder (629 mg, 98%). ^{1}H NMR (400 MHz, $CDCl_3$) δ 8.59 – 8.51 (m, 1H), 7.87 – 7.77 (m, 2H), 7.74 – 7.66 (m, 1H), 7.60 – 7.50 (m, 4H), 6.05 (s, 2H).

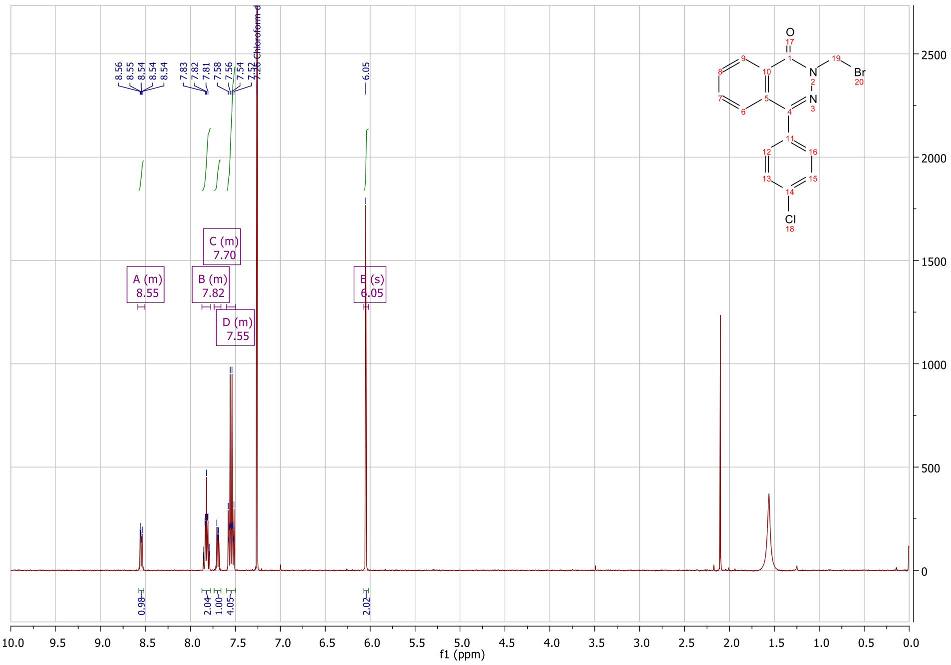

3-*O*-(4-(4-Chlorophenyl)phthalazin-1(2*H*)-one-2-ylmethyl)-D-galactal (7)

The compound was prepared according to general procedure D using intermediate **5b** (100 mg, 0.29 mmol), D-galactal (45 mg, 0.31 mmol), TBAI (120 mg, 0.32 mmol), K_2CO_3 (60 mg, 0.43 mmol) and dibutyltin oxide (80 mg, 0.32mmol). Column chromatography (4:1 EtOAc/Hex), followed by reversed-phase automated column chromatography gave compound **7** as a white solid (39 mg, 33% yield). ^{1}H NMR (400 MHz, $CDCl_3$) δ 8.58 – 8.50 (m, 1H), 7.87 – 7.78 (m, 2H), 7.74 – 7.67 (m, 1H), 7.57 – 7.49 (m, 4H), 6.46 (dd, *J* = 6.3, 1.7 Hz, 1H), 5.81 (d, *J* = 9.3 Hz, 1H), 5.67 (d, *J* = 9.3 Hz, 1H), 4.72 (dt, *J* = 6.3, 2.0 Hz, 1H), 4.56 – 4.51 (m, 1H), 4.32 – 4.26 (m, 1H), 4.05 (ddd, *J* = 11.4, 6.1, 3.4 Hz, 1H), 4.00 – 3.94 (m, 1H), 3.90 (ddd, *J* = 11.4, 8.7, 4.1 Hz, 1H), 3.57 (dd, *J* = 3.3, 1.1 Hz, 1H), 2.42 (dd, *J* = 8.7, 3.4 Hz, 1H). ^{13}C NMR (101 MHz, $CDCl_3$) δ 160.05, 147.13, 145.48, 135.73, 133.80, 133.02, 132.09, 130.77, 129.27, 129.03, 128.11, 127.64, 126.77, 99.18, 78.27, 76.04, 70.55, 64.16, 63.04. HPLC purity: 95.8%, HRMS calcd for $C_{21}H_{20}O_5N_2Cl + H^+$ (M + H^+): 415.1055, found: 415.1052.

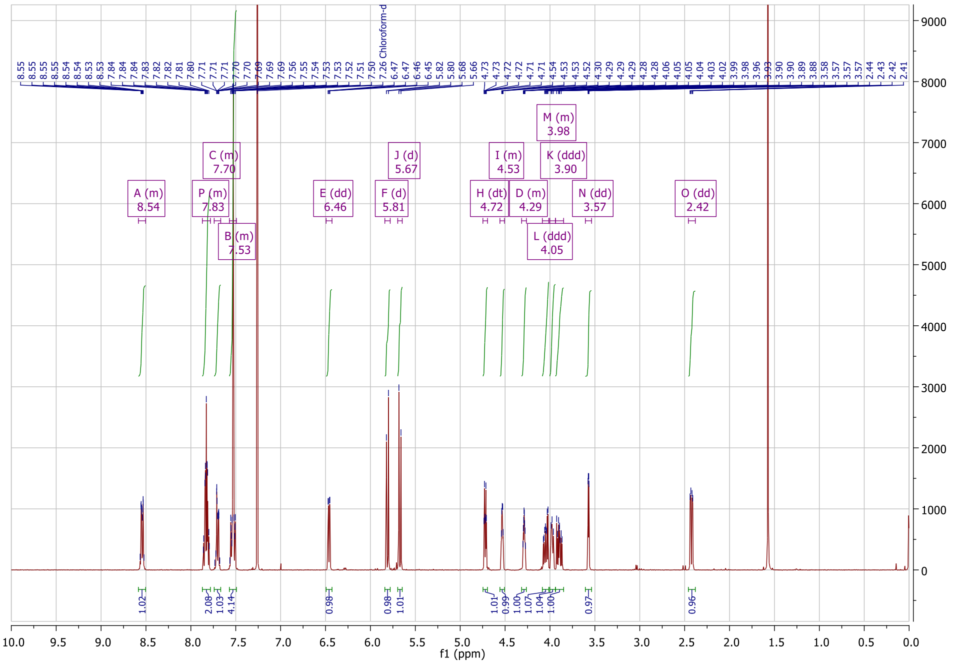

Compound 7. ^{13}C NMR (101 MHz, $CDCl_3$) δ 160.05, 147.13, 145.48, 135.73, 133.80, 133.02, 132.09, 130.77, 129.27, 129.03, 128.11, 127.64, 126.77, 99.18, 78.27, 76.04, 70.55, 64.16, 63.04.

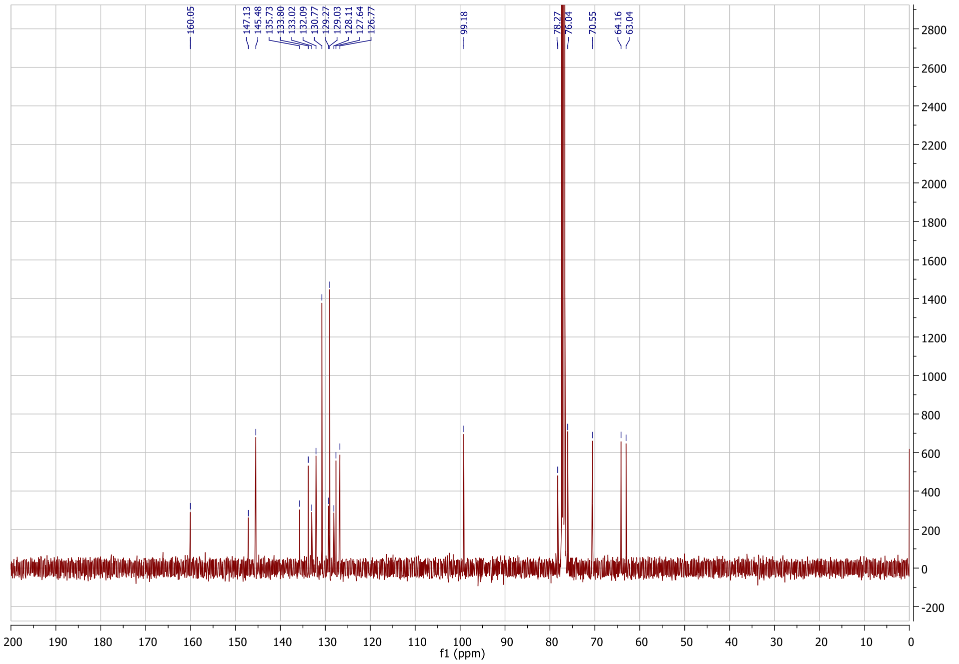

Chromatogram

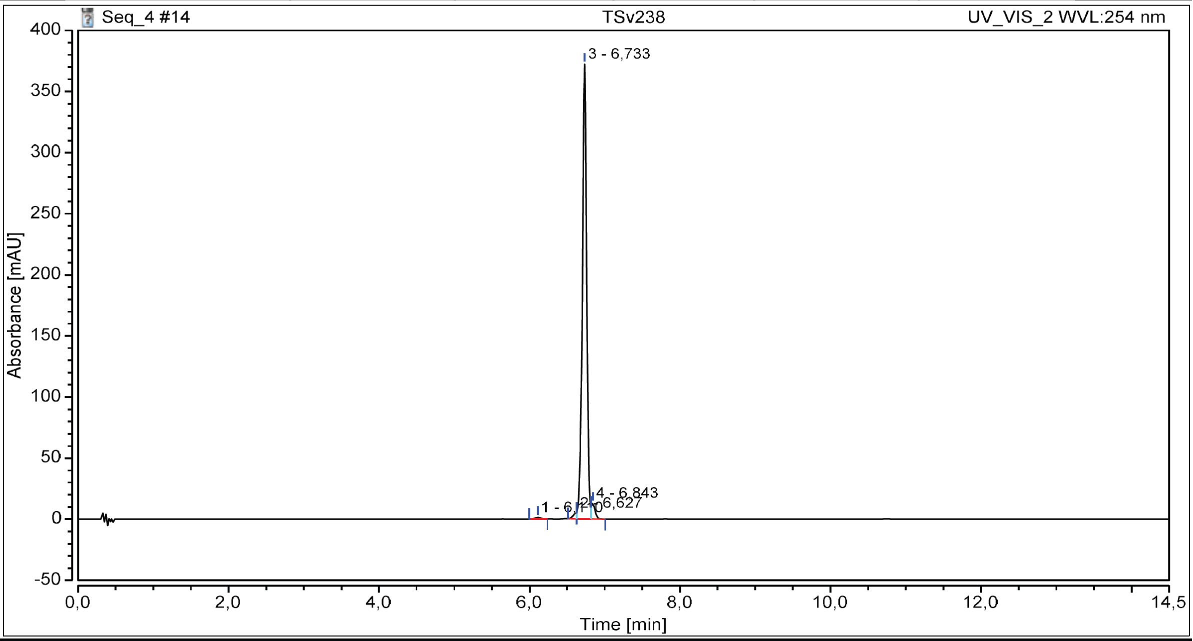

Integration Results

No.	Peak Name	Retention Time min	Area mAU*min	Height mAU	Relative Area %	Relative Height %	Amount n.a.
1		6,110	0,125	1,321	0,48	0,34	n.a.
2		6,627	0,242	4,718	0,94	1,21	n.a.
3		6,733	24,693	372,144	95,75	95,16	n.a.
4		6,843	0,728	12,887	2,82	3,30	n.a.
**Total:**			**25,788**	**391,070**	**100,00**	**100,00**	

4-(3,4-Dichlorophenyl)phthalazin-1(2*H*)-one (3c)

2-(3,4-Dichlorobenzoyl)benzoic acid (500 mg, 1.69 mmol) was dissolved in ethanol (10 mL). Hydrazine hydrate (0.250 mL, 5.08 mmol) was added and the mixture was refluxed for 4 h. The mixture was allowed to cool, solids were filtered off, washed with EtOH, and dried *in vacuo* to give intermediate **3c** as a light-pink solid (408 mg, 83%). ^{1}H NMR (400 MHz, DMSO) δ 12.95 (s, 1H), 8.38 – 8.31 (m, 1H), 7.96 – 7.86 (m, 3H), 7.83 (d, *J* = 8.3 Hz, 1H), 7.72 – 7.65 (m, 1H), 7.61 (dd, *J* = 8.3, 2.1 Hz, 1H).

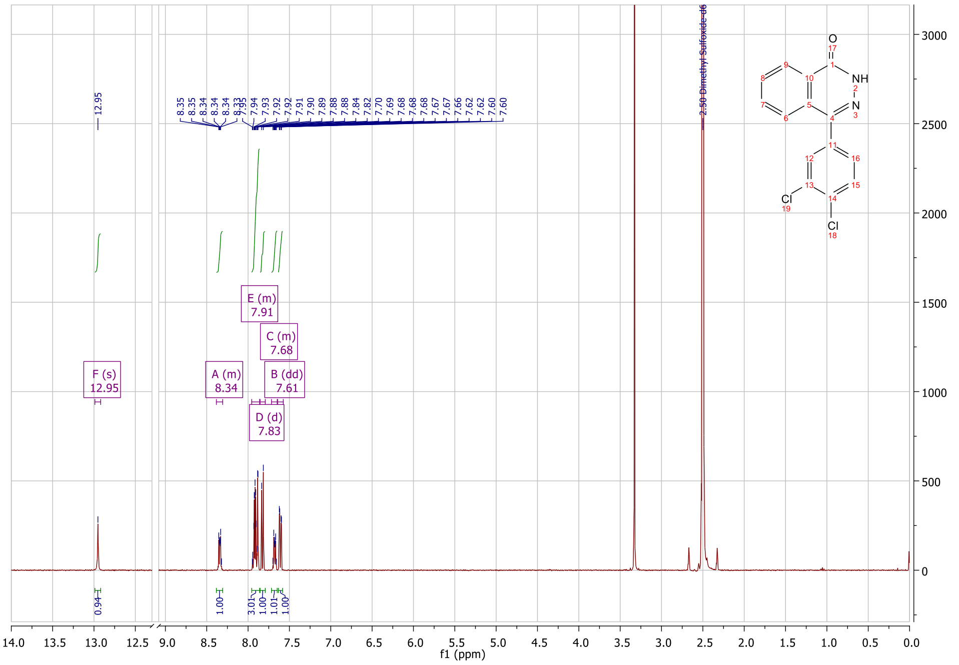

(4-(3,4-Dichlorophenyl)phthalazin-1(2*H*)-one-2-yl)methyl pivalate (4c)

The compound was prepared according to general procedure B2 using intermediate **3c** (200 mg, 0.69 mmol) to give the intermediate **4c** as a light pink solid (268 mg, 96% yield). ^{1}H NMR (400 MHz, $CDCl_3$) δ 8.59 – 8.53 (m, 1H), 7.86 – 7.80 (m, 2H), 7.72 – 7.65 (m, 2H), 7.62 (d, *J* = 8.2 Hz, 1H), 7.45 (dd, *J* = 8.2, 2.1 Hz, 1H), 6.18 (s, 2H), 1.22 (s, 9H).

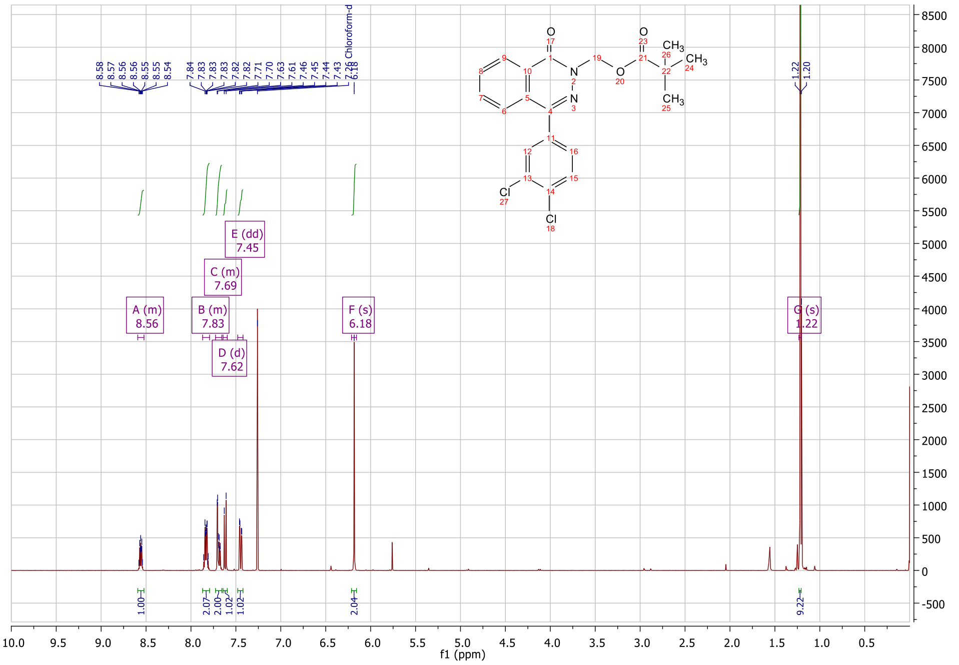

2-(Bromomethyl)-4-(3,4-dichlorophenyl)phthalazin-1(2*H*)-one (5c)

The compound was prepared according to general procedure C using intermediate **4c** (260 mg, 0.64 mmol) to give intermediate **5c** as a light pink powder (195 mg, 79%). ^{1}H NMR (400 MHz, $CDCl_3$) δ 8.60 – 8.50 (m, 1H), 7.89 – 7.80 (m, 2H), 7.73 (d, *J* = 2.0 Hz, 1H), 7.72 – 7.66 (m, 1H), 7.63 (d, *J* = 8.3 Hz, 1H), 7.47 (dd, *J* = 8.3, 2.0 Hz, 1H), 6.04 (s, 2H).

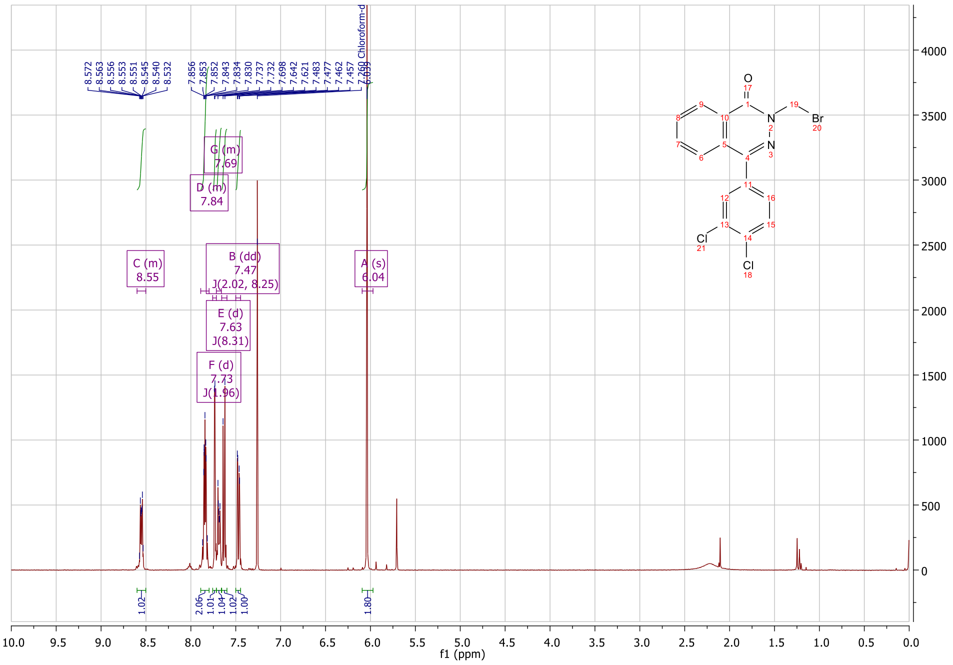

3-*O*-(4-(3,4-Dichlorophenyl)phthalazin-1(2*H*)-one-2-ylmethyl)-D-galactal (8)

The compound was prepared according to general procedure D using intermediate **5c** (80 mg, 0.21 mmol), D-galactal (34 mg, 0.23 mmol), TBAI (84 mg, 0.23 mmol), K_2CO_3 (45 mg, 0.33 mmol) and dibutyltin oxide (60 mg, 0.24 mmol). Column chromatography (4:1 EtOAc/Hex), followed by reversed-phase automated column chromatography gave compound **8** as a white solid (17 mg, 18% yield). ^{1}H NMR (400 MHz, $CDCl_3$) δ 8.58 – 8.52 (m, 1H), 7.88 – 7.81 (m, 2H), 7.74 – 7.67 (m, 2H), 7.62 (d, *J* = 8.2 Hz, 1H), 7.45 (dd, *J* = 8.2, 2.1 Hz, 1H), 6.47 (dd, *J* = 6.3, 1.6 Hz, 1H), 5.80 (d, *J* = 9.3 Hz, 1H), 5.67 (d, *J* = 9.3 Hz, 1H), 4.72 (dt, *J* = 6.3, 2.0 Hz, 1H), 4.56 – 4.51 (m, 1H), 4.32 – 4.26 (m, 1H), 4.05 (dd, *J* = 11.3, 6.0 Hz, 1H), 4.00 – 3.95 (m, 1H), 3.90 (dd, *J* = 11.1, 3.5 Hz, 1H), 3.51 (s, 1H), 2.38 (s, 1H). ^{13}C NMR (101 MHz, $CDCl_3$) δ 159.96, 145.86, 145.51, 134.47, 134.01, 133.98, 133.19, 132.27, 131.36, 130.74, 128.96, 128.69, 128.09, 127.78, 126.45, 99.13, 78.30, 76.04, 70.64, 64.15, 63.02. HPLC purity: 98.9%, HRMS calcd for $C_{21}H_{19}O_5N_2Cl_2 + H^+$ ($M + H^+$): 449.0666, found: 449.0662.

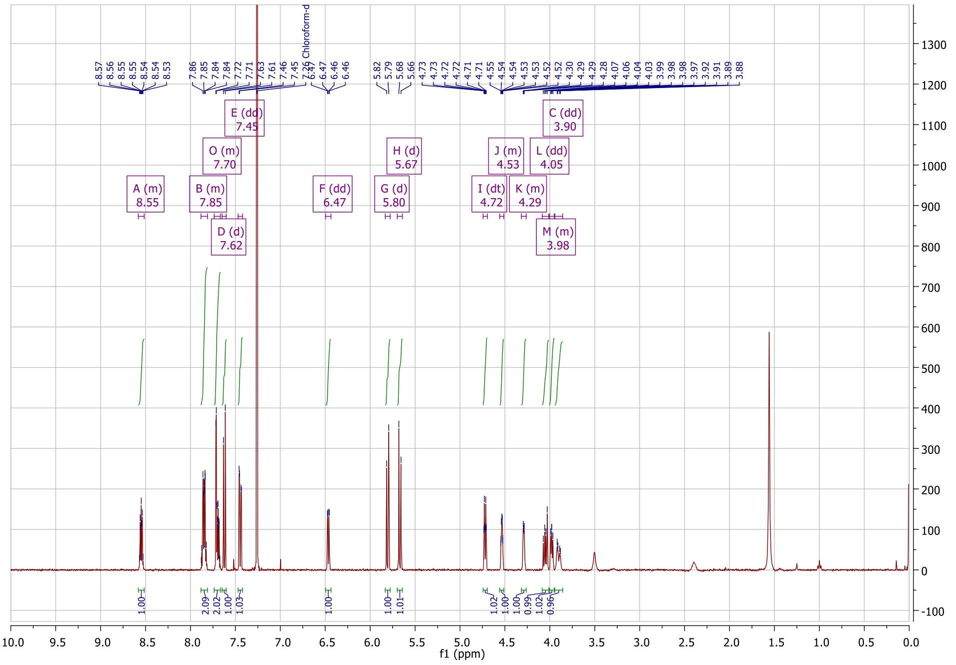

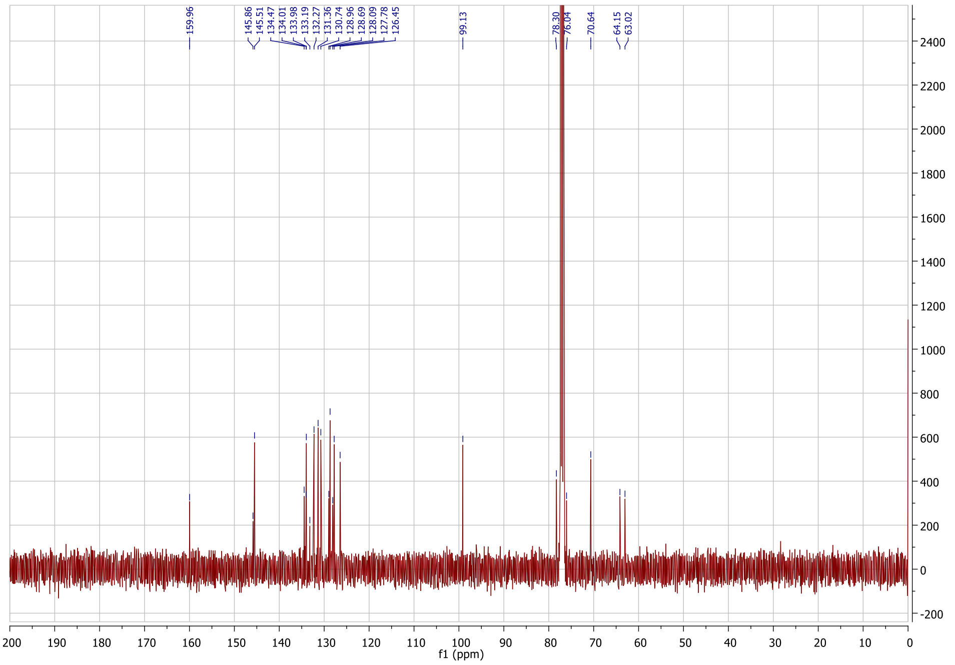

Chromatogram

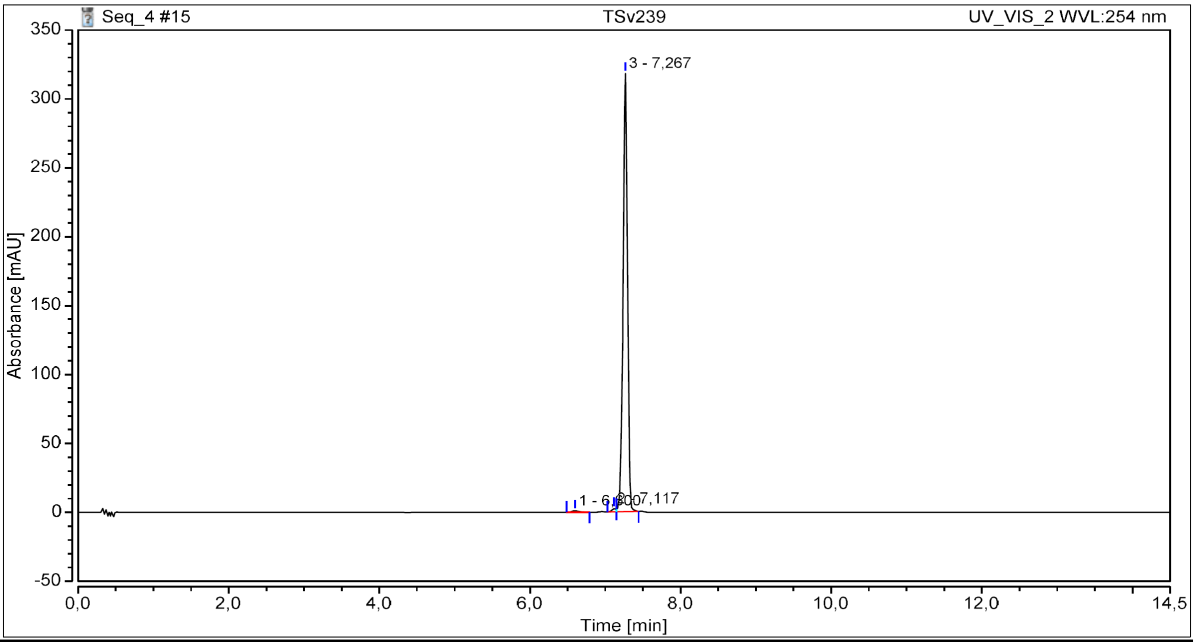

Integration Results

No.	Peak Name	Retention Time min	Area mAU*min	Height mAU	Relative Area %	Relative Height %	Amount n.a.
1		6,600	0,108	1,039	0,49	0,32	n.a.
2		7,117	0,144	2,386	0,66	0,74	n.a.
3		7,267	21,603	317,974	98,85	98,93	n.a.
**Total:**			**21,855**	**321,399**	**100,00**	**100,00**	

(4-(4-Methylphenyl)phthalazin-1(2*H*)-one-2-yl)methyl pivalate (4d)

The compound was prepared according to general procedure B1 starting from 4-(4-methylphenyl)-phthalazin-1(2*H*)-one **3d** (150 mg, 0.63 mmol). Column chromatography (1:2 EtOAc/Hept.) gave intermediate **4d** as a white powder (201 mg, 90% yield). ^{1}H NMR (400 MHz, $CDCl_3$) δ 8.58 – 8.51 (m, 1H), 7.83 – 7.72 (m, 3H), 7.51 – 7.43 (m, 2H), 7.34 (d, *J* = 7.8 Hz, 4H), 6.19 (s, 2H), 2.46 (s, 3H), 1.22 (s, 9H).

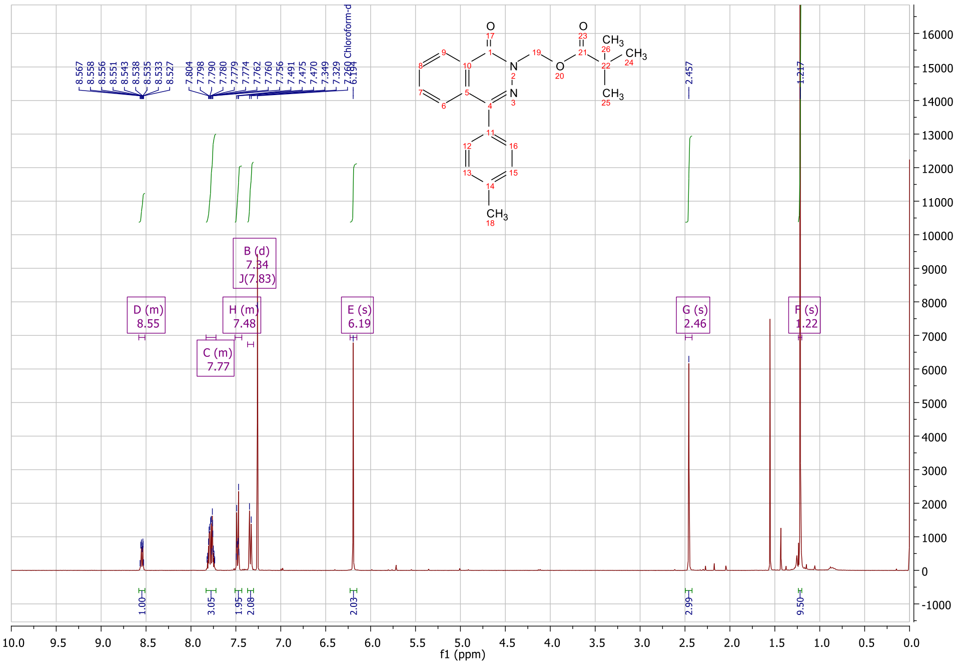

2-(Bromomethyl)-4-(4-methylphenyl)phthalazin-1(2*H*)-one (5d)

The compound was prepared according to general procedure C using intermediate **4d** (100 mg, 0.29 mmol) to give intermediate **5d** as a white powder (78 mg, 83%). ^{1}H NMR (400 MHz, $CDCl_3$) δ 8.56 – 8.52 (m, 1H), 7.83 – 7.73 (m, 3H), 7.53 – 7.48 (m, 2H), 7.35 (d, *J* = 7.8 Hz, 2H), 6.06 (s, 2H), 2.46 (s, 3H).

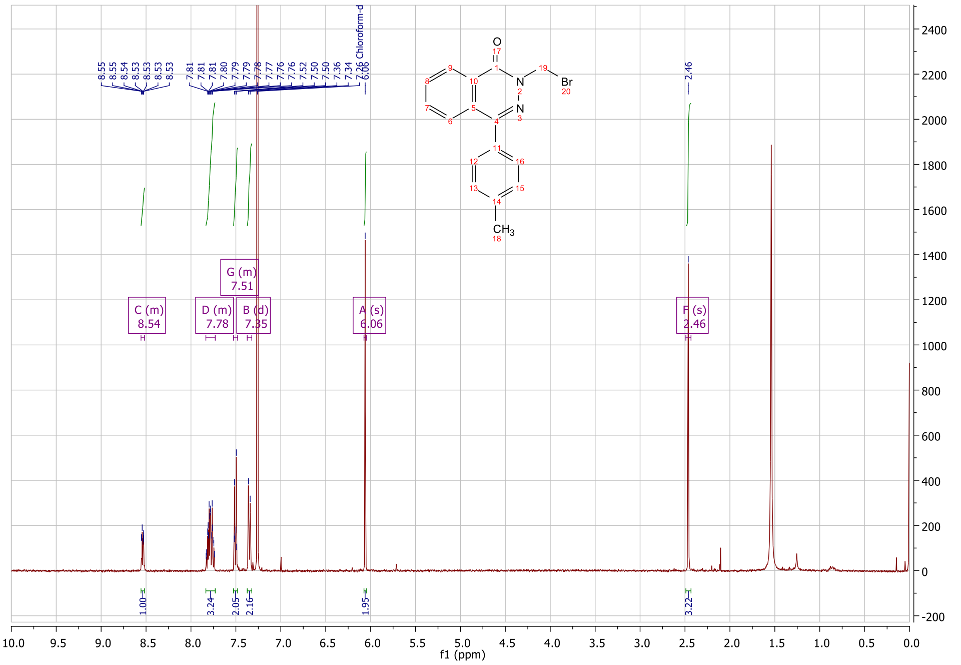

3-*O*-(4-(4-Methylphenyl)phthalazin-1(2*H*)-one-2-ylmethyl)-D-galactal (9)

The compound was prepared according to general procedure D using intermediate **5d** (75 mg, 0.209 mmol), D-galactal (40 mg, 0.274 mmol), TBAI (97 mg, 0.265 mmol), K_2CO_3 (55 mg, 398 mmol) and dibutyltin oxide (72 mg, 0.289 mmol). Column chromatography (1:20 MeOH/DCM), followed by reversed-phase automated column chromatography gave product **9** as a white solid (32 mg, 36% yield). ^{1}H NMR (400 MHz, $CDCl_3$) δ 8.56 – 8.51 (m, 1H), 7.85 – 7.73 (m, 3H), 7.50 – 7.45 (m, 2H), 7.34 (d, *J* = 7.8 Hz, 2H), 6.46 (dd, *J* = 6.3, 1.6 Hz, 1H), 5.81 (d, *J* = 9.3 Hz, 1H), 5.68 (d, *J* = 9.3 Hz, 1H), 4.73 (dt, *J* = 6.3, 2.0 Hz, 1H), 4.56 – 4.50 (m, 1H), 4.31 – 4.26 (m, 1H), 4.05 (ddd, *J* = 11.4, 6.1, 3.3 Hz, 1H), 3.99 – 3.94 (m, 1H), 3.89 (ddd, *J* = 11.5, 8.8, 4.0 Hz, 1H), 3.64 (dd, *J* = 3.2, 1.0 Hz, 1H), 2.49 – 2.43 (m, 4H). ^{13}C NMR (101 MHz, $CDCl_3$) δ 160.30, 148.50, 145.54, 139.68, 133.73, 131.97, 131.83, 129.77, 129.54, 129.43, 128.23, 127.58, 127.35, 99.42, 78.42, 77.16, 76.17, 70.61, 64.31, 63.17, 21.52. HPLC purity: 95.7%, HRMS calcd for $C_{22}H_{23}O_5N_2 + H^+$ ($M + H^+$): 395.16015, found: 395.16051.

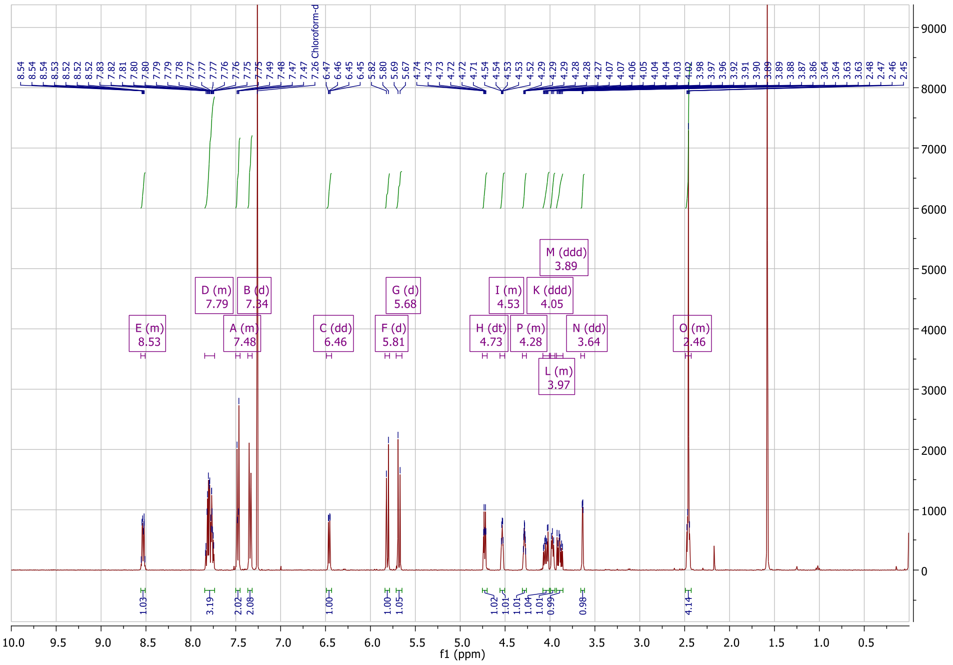

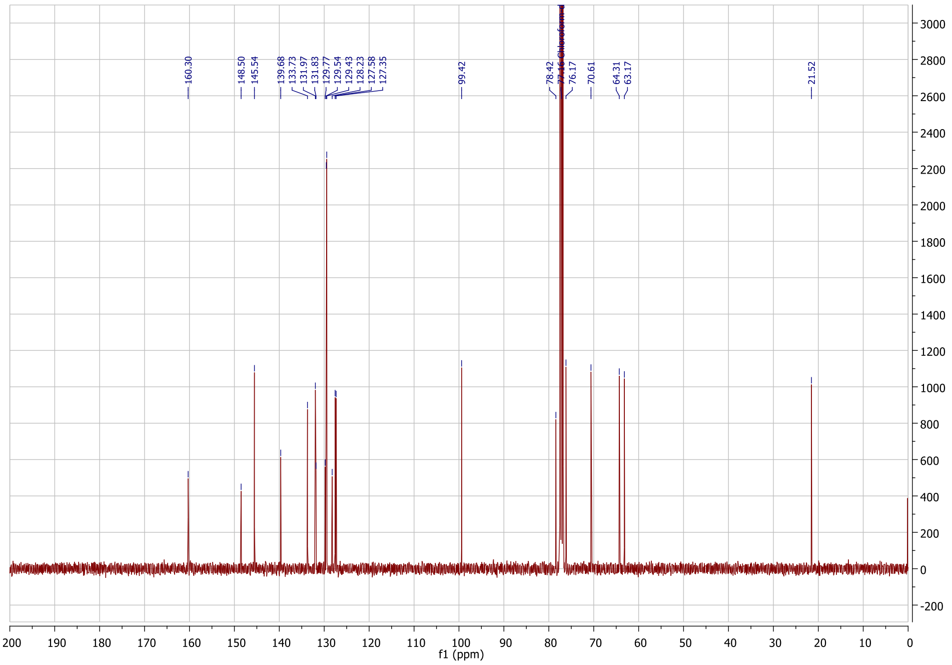

Chromatogram

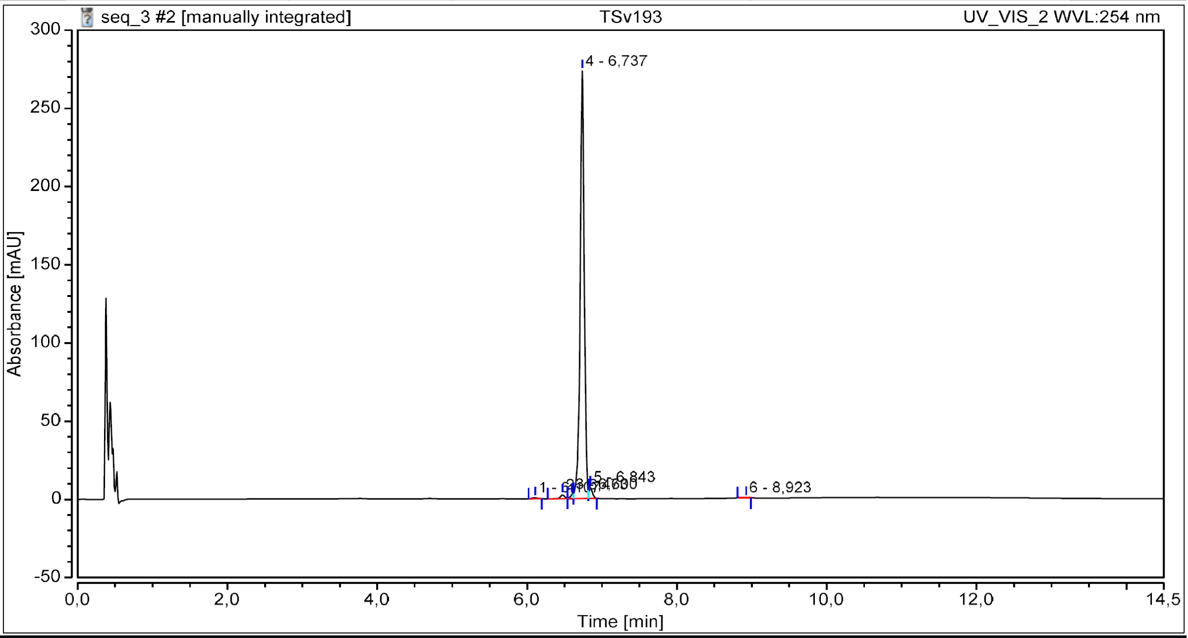

Integration Results

No.	Peak Name	Retention Time min	Area mAU*min	Height mAU	Relative Area %	Relative Height %	Amount n.a.
1		6,107	0,059	0,686	0,33	0,24	n.a.
2		6,473	0,163	2,525	0,91	0,88	n.a.
3		6,600	0,121	2,783	0,68	0,97	n.a.
4		6,737	17,085	273,487	95,71	95,27	n.a.
5		6,843	0,396	7,293	2,22	2,54	n.a.
6		8,923	0,027	0,287	0,15	0,10	n.a.
**Total:**			**17,851**	**287,060**	**100,00**	**100,00**	

(4-(4-Bromophenyl)phthalazin-1(2*H*)-one-2-yl)methyl pivalate (4e)

The compound was prepared according to general procedure B1 starting from 4-(4-bromophenyl)-phthalazin-1(2*H*)-one **3e** (200 mg, 0.66 mmol) to give intermediate **4e** as a white powder (178 mg, 65% yield). ^{1}H NMR (400 MHz, $CDCl_3$) δ 8.61 – 8.50 (m, 1H), 7.86 – 7.76 (m, 2H), 7.73 – 7.63 (m, 3H), 7.51 – 7.43 (m, 2H), 6.19 (s, 2H), 1.21 (s, 9H).

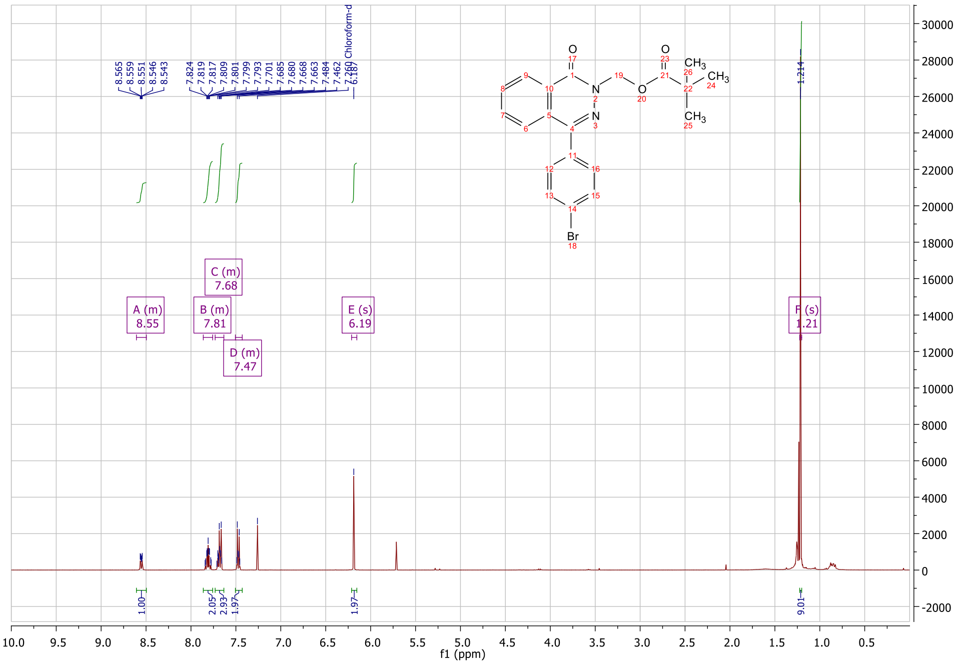

2-(Bromomethyl)-4-(4-bromophenyl)phthalazin-1(2*H*)-one (5e)

The compound was prepared according to general procedure C using intermediate **4e** (120 mg, 0.29 mmol) to give intermediate **5e** as a white powder (104 mg, 91%). ^{1}H NMR (400 MHz, $CDCl_3$) δ 8.59 – 8.50 (m, 1H), 7.88 – 7.76 (m, 2H), 7.74 – 7.63 (m, 3H), 7.53 – 7.44 (m, 2H), 6.05 (s, 2H).

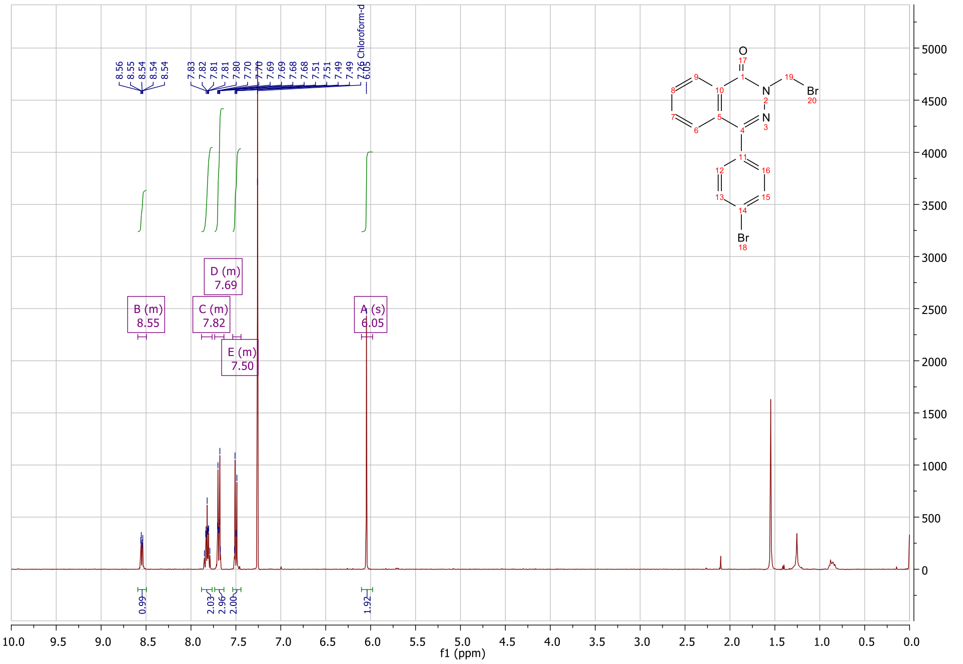

3-*O*-(4-(4-Bromophenyl)phthalazin-1(2*H*)-one-2-ylmethyl)-D-galactal (10)

The compound was prepared according to general procedure D using intermediate **5e** (50 mg, 0.143 mmol), D-galactal (22 mg, 0.151 mmol), TBAI (54 mg, 0.146 mmol), K_2CO_3 (30 mg, 0.217 mmol) and dibutyltin oxide (40 mg, 0.161 mmol). Column chromatography (1:20 MeOH/DCM), followed by reversed-phase automated column chromatography gave product **10** as a white solid (26 mg, 40% yield). ^{1}H NMR (400 MHz, $CDCl_3$) δ 8.54 (d, *J* = 7.7 Hz, 1H), 7.87 – 7.78 (m, 2H), 7.74 – 7.64 (m, 3H), 7.47 (d, *J* = 7.9 Hz, 2H), 6.45 (d, *J* = 6.3 Hz, 1H), 5.80 (d, *J* = 9.3 Hz, 1H), 5.67 (d, *J* = 9.3 Hz, 1H), 4.77 – 4.68 (m, 1H), 4.57 – 4.51 (m, 1H), 4.28 (s, 1H), 4.08 – 4.00 (m, 1H), 4.00 – 3.94 (m, 1H), 3.94 – 3.84 (m, 1H), 3.58 (d, *J* = 2.7 Hz, 1H), 2.49 (dd, *J* = 8.5, 3.0 Hz, 1H). ^{13}C NMR (101 MHz, $CDCl_3$) δ 160.30, 147.42, 145.72, 134.07, 133.74, 132.35, 132.23, 131.28, 129.45, 128.35, 127.90, 127.00, 124.20, 99.43, 78.53, 77.16, 76.30, 70.82, 64.37, 63.23. HPLC purity: 96.7%, HRMS calcd for $C_{21}H_{20}O_5N_2Br + H^+$ (M + H^+): 459.05501, found: 459.05511.

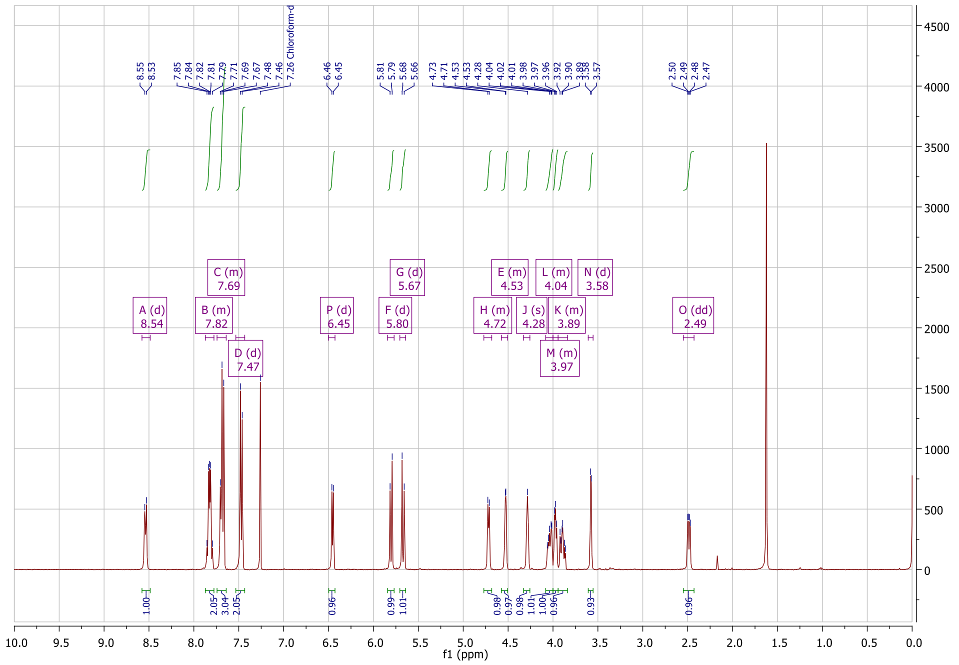

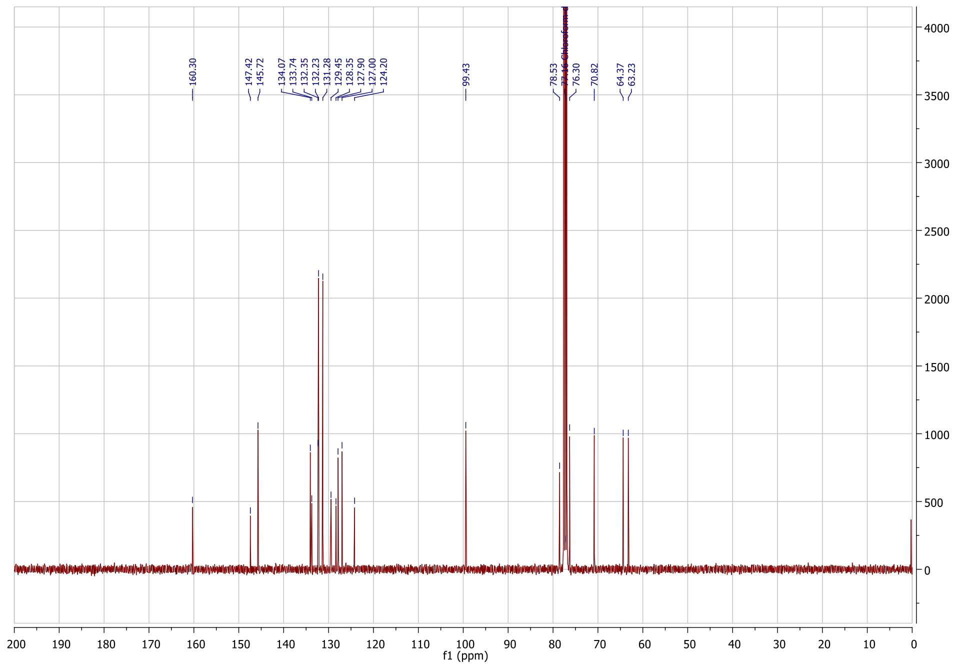

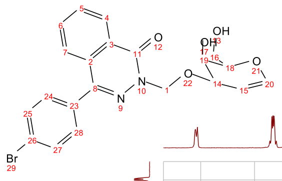
OH
OH
O
O
N
N
O
Br

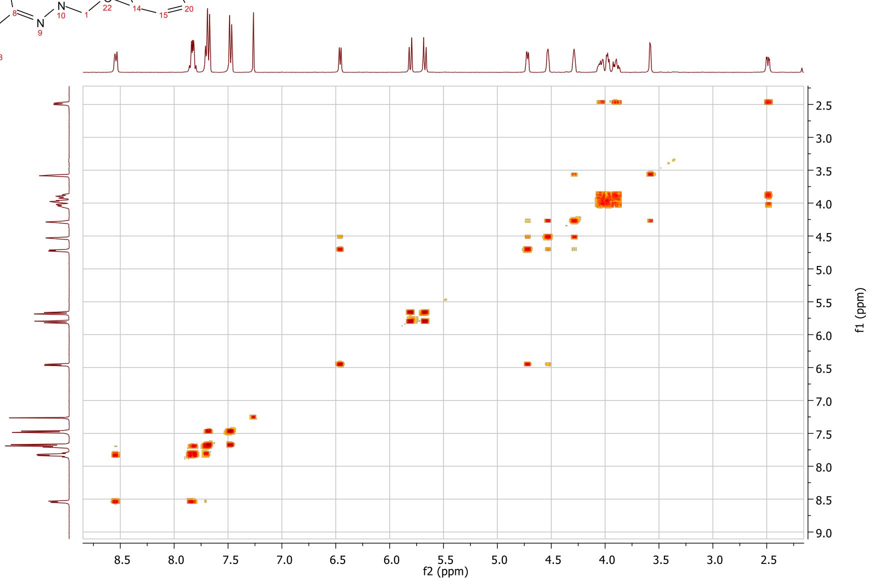
f2 (ppm)
f1 (ppm)

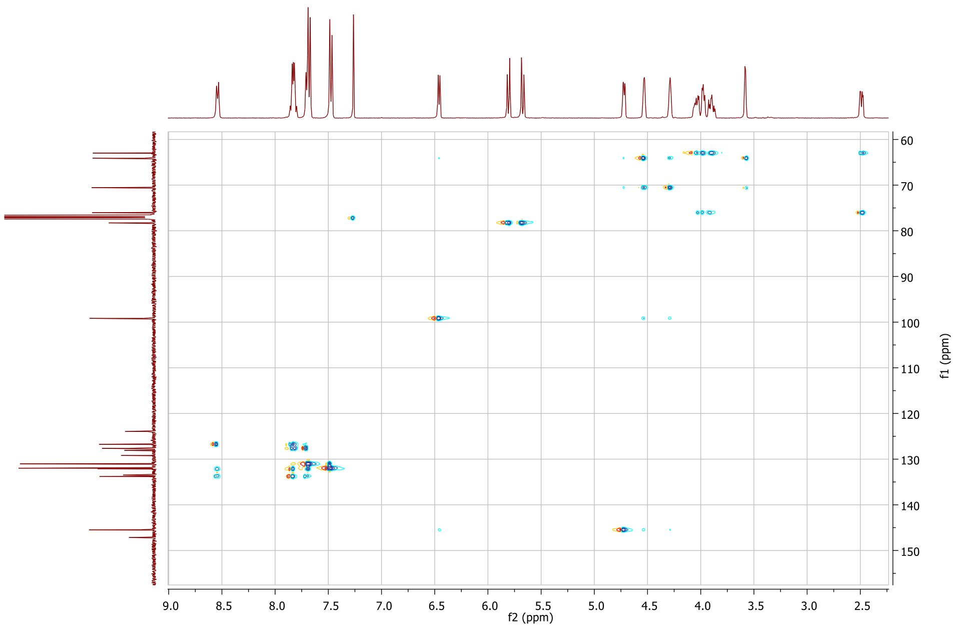
f2 (ppm)
f1 (ppm)

Chromatogram

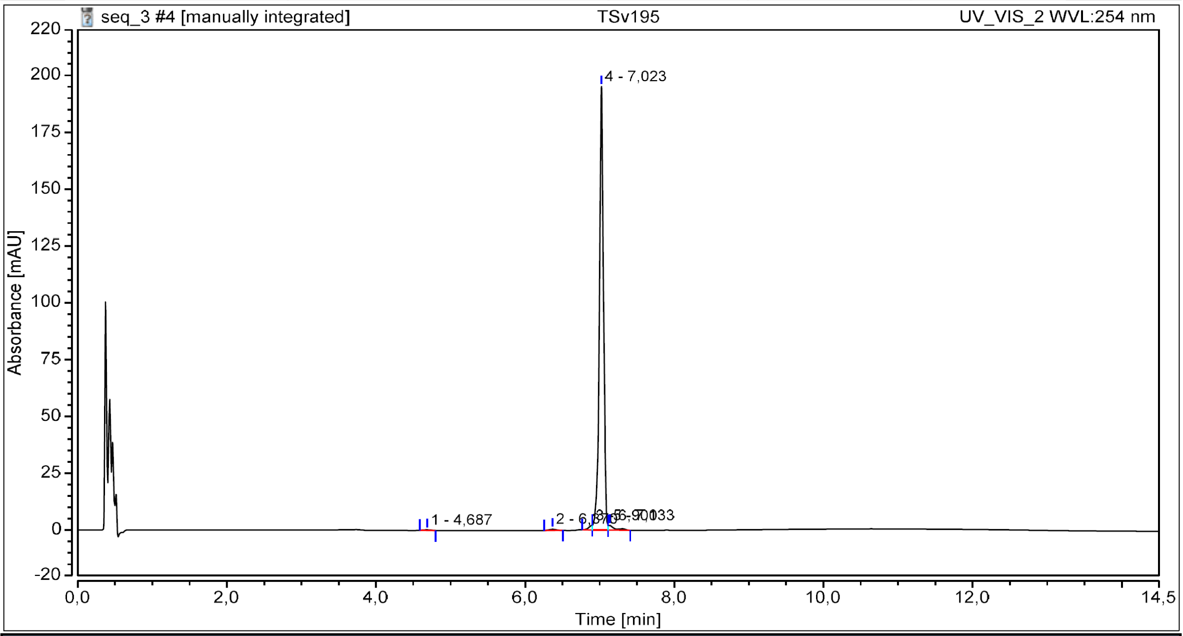

Integration Results

No.	Peak Name	Retention Time min	Area mAU*min	Height mAU	Relative Area %	Relative Height %	Amount n.a.
1		4,687	0,028	0,245	0,22	0,12	n.a.
2		6,370	0,059	0,572	0,46	0,29	n.a.
3		6,900	0,105	2,000	0,82	1,00	n.a.
4		7,023	12,404	195,084	96,73	97,52	n.a.
5		7,133	0,226	2,134	1,76	1,07	n.a.
**Total:**			**12,823**	**200,035**	**100,00**	**100,00**	

References

(1) Hassan, M.; Van Klaveren, S.; Håkansson, M.; Diehl, C.; Kovačič, R.; Baussière, F.; Sundin, A. P.; Dernovšek, J.; Walse, B.; Zetterberg, F.; Leffler, H.; Anderluh, M.; Tomašič, T.; Jakopin, Ž.; Nilsson, U. J. Benzimidazole–Galactosides Bind Selectively to the Galectin-8 N-Terminal Domain: Structure-Based Design and Optimisation. *Eur. J. Med. Chem.* **2021**, *223*, 113664. https://doi.org/10.1016/J.EJMECH.2021.113664.

(2) Winter, G.; Waterman, D. G.; Parkhurst, J. M.; Brewster, A. S.; Gildea, R. J.; Gerstel, M.; Fuentes-Montero, L.; Vollmar, M.; Michels-Clark, T.; Young, I. D.; Sauter, N. K.; Evans, G. DIALS: Implementation and Evaluation of a New Integration Package. *Acta Crystallogr. Sect. D Struct. Biol.* **2018**, *74*, 85–97. https://doi.org/10.1107/S2059798317017235.

(3) Evans, P. R.; Murshudov, G. N. How Good Are My Data and What Is the Resolution? *Acta Crystallogr. Sect. D Biol. Crystallogr.* **2013**, *69* (7), 1204–1214. https://doi.org/10.1107/S0907444913000061.

(4) Murshudov, G. N.; Skubák, P.; Lebedev, A. A.; Pannu, N. S.; Steiner, R. A.; Nicholls, R. A.; Winn, M. D.; Long, F.; Vagin, A. A. REFMAC5 for the Refinement of Macromolecular Crystal Structures. *Acta Crystallogr. Sect. D Biol. Crystallogr.* **2011**, *67*, 355–367. https://doi.org/10.1107/S0907444911001314.

(5) Emsley, P.; Lohkamp, B.; Scott, W. G.; Cowtan, K. Features and Development of Coot. *Acta Crystallogr. Sect. D Biol. Crystallogr.* **2010**, *66*, 486–501. https://doi.org/10.1107/S0907444910007493.

(6) Chen, V. B.; Arendall, W. B.; Headd, J. J.; Keedy, D. A.; Immormino, R. M.; Kapral, G. J.; Murray, L. W.; Richardson, J. S.; Richardson, D. C. MolProbity: All-Atom Structure Validation for Macromolecular Crystallography. *Acta Crystallogr. Sect. D Biol. Crystallogr.* **2010**, *66*, 12–21. https://doi.org/10.1107/S0907444909042073.

(7) van Klaveren, S.; Sundin, A. P.; Jakopin, Ž.; Anderluh, M.; Leffler, H.; Nilsson, U. J.; Tomašič, T. Selective Galectin-8N Ligands: The Design and Synthesis of Phthalazinone- <scp>d</Scp> -Galactals. *ChemMedChem* **2022**, *17* (6), e202100575. https://doi.org/10.1002/cmdc.202100575.

(8) Chen, X.; Murawski, A.; Patel, K.; Crespi, C. L.; Balimane, P. V. A Novel Design of Artificial Membrane for Improving the PAMPA Model. *Pharm. Res.* **2008**, *25* (7), 1511–1520. https://doi.org/10.1007/s11095-007-9517-8.

(9) Balimane, P. V; Han, Y. H.; Chong, S. Current Industrial Practices of Assessing Permeability and P-Glycoprotein Interaction. *AAPS J.* **2006**, *8* (1), 1. https://doi.org/10.1208/aapsj080101.

(10) Terán, C.; Besada, P.; Vila, N.; Costas-Lago, M. C. Recent Advances in the Synthesis of Phthalazin-1(2H)-One Core as a Relevant Pharmacophore in Medicinal Chemistry. *Eur. J. Med. Chem.* **2019**, *161*, 468–478. https://doi.org/10.1016/j.ejmech.2018.10.047.

(11) Court, J. J.; Lessen, T. A.; Hlasta, D. J. A Novel and Practical Method for the N-Bromomethylation of Imides and Isothiazolone 1,1-Dioxides. *Synlett* **1995**, *5*, 423–424. https://doi.org/10.1055/s-1995-4994.

(12) Ballell, L.; Joosten, J. A. F.; Maate, F. A. el; Liskamp, R. M. J.; Pieters, R. J. Microwave-Assisted, Tin-Mediated, Regioselective 3-O-Alkylation of Galactosides. *Tetrahedron Lett.* **2004**, *45* (35), 6685–6687. https://doi.org/10.1016/J.TETLET.2004.06.113.

(13) Carlsson, S.; Öberg, C. T.; Carlsson, M. C.; Sundin, A.; Nilsson, U. J.; Smith, D.; Cummings, R. D.; Almkvist, J.; Karlsson, A.; Leffler, H. Affinity of Galectin-8 and Its Carbohydrate Recognition Domains for Ligands in Solution and at the Cell Surface. *Glycobiology* **2007**, *17* (6), 663–676. https://doi.org/10.1093/glycob/cwm026.

(14) Delaine, T.; Collins, P.; MacKinnon, A.; Sharma, G.; Stegmayr, J.; Rajput, V. K.; Mandal, S.; Cumpstey, I.; Larumbe, A.; Salameh, B. A.; Kahl-Knutsson, B.; van Hattum, H.; van Scherpenzeel, M.; Pieters, R. J.; Sethi, T.; Schambye, H.; Oredsson, S.; Leffler, H.; Blanchard, H.; Nilsson, U. J. Galectin-3-Binding Glycomimetics That Strongly Reduce Bleomycin-Induced Lung Fibrosis and Modulate Intracellular Glycan Recognition. *ChemBioChem* **2016**, *17* (18), 1759–1770. https://doi.org/10.1002/cbic.201600285.

(15) Sörme, P.; Kahl-Knutsson, B.; Huflejt, M.; Nilsson, U. J.; Leffler, H. Fluorescence Polarization as an Analytical Tool to Evaluate Galectin-Ligand Interactions. *Anal. Biochem.* **2004**, *334* (1), 36–47. https://doi.org/10.1016/j.ab.2004.06.042.
